# Supplementary material for: Genomic variation associated with cardiovascular disease progression following preeclampsia: a systematic review
Source: Front Epidemiol. 2023 Oct 16;3:1221222. doi: 10.3389/fepid.2023.1221222 (PMC10911037; doi:10.3389/fepid.2023.1221222)
Supplement: Supplementary file 1 [file Datasheet1.docx]

***Supplementary Material***

**Genomic variation associated with cardiovascular disease progression following preeclampsia: A systematic review**

**Gayathry Krishnamurthy^1^, Phuong Tram Nguyen^1^, Bao Ngoc Tran^2^, Hoang T Phan^1^, Shaun P. Brennecke^3,4^, Eric K Moses^1^, Phillip E. Melton^1,5*^**

^1^Menzies Institute for Medical Research, The University of Tasmania, Hobart, TAS, Australia.

^2^Wicking Dementia Research and Education Center, The University of Tasmania, Hobart, TAS, Australia.

^3^Pregnancy Research Centre, Department of Maternal‐Fetal Medicine, The Royal Women’s Hospital, Melbourne, VIC, Australia.

^4^Department of Obstetrics and Gynaecology, The Royal Women’s Hospital, The University of Melbourne, Melbourne, VIC, Australia.

^5^School of Global and Population Health, The University of Western Australia, Crawley, WA, Australia.

***Correspondence**:

Gayathry Krishnamurthy

[gayathry.krishnamurthy@utas.edu.au](mailto:gayathry.krishnamurthy@utas.edu.au)

| TABLE OF CONTENTS | |  |
| --- | --- | --- |
| Supplementary Table | **Contents** | **Page number** |
| 1  2  3  4  5  6  7  8  9  10  11 | PRISMA 2020 CHECKLIST………………………………  PICO SEARCH STRATEGY……………………………...  SEARCH STRATEGY (27/08/2021)……………………....  UPDATED SEARCH (02/02/2023)………………………..  UPDATED SEARCH (28/07/2023)………………………..  Quality Assessment Scores using the Newcastle-Ottawa Scale for Case-control Studies……………………………...  Quality Assessment Scores using the Newcastle-Ottawa Scale for Cohort Studies………………………………........  Quality Assessment Scores using the Newcastle-Ottawa Scale for Cross-sectional Studies…………………………..  Quality Assessment using the Newcastle-Ottawa Scale for Case-control Studies (Detailed Description)……………….  Quality Assessment using the Newcastle-Ottawa Scale for Cohort Studies (Detailed Description)……………………..  Quality Assessment using the Newcastle-Ottawa Scale for Cross-sectional Studies (Detailed Description)……………. | 3-6  6-8  9-19  20-30  31-40  40-48  49-50  51  51-61  62-64  64 |


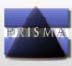


**SUPPLEMENTARY TABLE 1: PRISMA 2020 CHECKLIST**

| **Section and Topic** | **Item #** | **Checklist item** | **Reported on page #** |
| --- | --- | --- | --- |
| **TITLE** | | |  |
| Title | 1 | Identify the report as a systematic review. | 1 |
| **ABSTRACT** | | |  |
| Abstract | 2 | See the PRISMA 2020 for Abstracts checklist. | 1, 2 |
| **INTRODUCTION** | | |  |
| Rationale | 3 | Describe the rationale for the review in the context of existing knowledge. | 2,3 |
| Objectives | 4 | Provide an explicit statement of the objective(s) or question(s) the review addresses. | 3 |
| **METHODS** | | |  |
| Eligibility criteria | 5 | Specify the inclusion and exclusion criteria for the review and how studies were grouped for the syntheses. | 3,4 |
| Information sources | 6 | Specify all databases, registers, websites, organisations, reference lists and other sources searched or consulted to identify studies. Specify the date when each source was last searched or consulted. | 3,4 |
| Search strategy | 7 | Present the full search strategies for all databases, registers and websites, including any filters and limits used. | 3,4, Supplementary tables 2-5 |
| Selection process | 8 | Specify the methods used to decide whether a study met the inclusion criteria of the review, including how many reviewers screened each record and each report retrieved, whether they worked independently, and if applicable, details of automation tools used in the process. | 3,4 |
| Data collection process | 9 | Specify the methods used to collect data from reports, including how many reviewers collected data from each report, whether they worked independently, any processes for obtaining or confirming data from study investigators, and if applicable, details of automation tools used in the process. | 3,4 |
| Data items | 10a | List and define all outcomes for which data were sought. Specify whether all results that were compatible with each outcome domain in each study were sought (e.g. for all measures, time points, analyses), and if not, the methods used to decide which results to collect. | Table 1 and 2 |
|  | 10b | List and define all other variables for which data were sought (e.g. participant and intervention characteristics, funding sources). Describe any assumptions made about any missing or unclear information. | Table 1 and 2 |
| Study risk of bias assessment | 11 | Specify the methods used to assess risk of bias in the included studies, including details of the tool(s) used, how many reviewers assessed each study and whether they worked independently, and if applicable, details of automation tools used in the process. | 5,6 |
| Effect measures | 12 | Specify for each outcome the effect measure(s) (e.g. risk ratio, mean difference) used in the synthesis or presentation of results. | Table 3 and 4 |
| Synthesis methods | 13a | Describe the processes used to decide which studies were eligible for each synthesis (e.g. tabulating the study intervention characteristics and comparing against the planned groups for each synthesis (item #5)). | 3-5, Table 1 and 2 |
|  | 13b | Describe any methods required to prepare the data for presentation or synthesis, such as handling of missing summary statistics, or data conversions. | Not applicable |
|  | 13c | Describe any methods used to tabulate or visually display results of individual studies and syntheses. | Table 3 and 4 |
|  | 13d | Describe any methods used to synthesise results and provide a rationale for the choice(s). If meta-analysis was performed, describe the model(s), method(s) to identify the presence and extent of statistical heterogeneity, and software package(s) used. | Not applicable |
|  | 13e | Describe any methods used to explore possible causes of heterogeneity among study results (e.g. subgroup analysis, meta-regression). | Not applicable |
|  | 13f | Describe any sensitivity analyses conducted to assess robustness of the synthesised results. | Not applicable |
| Reporting bias/ Quality assessment | 14 | Describe any methods used to assess risk of bias or Quality assessment. | 5,6 |
| Certainty assessment | 15 | Describe any methods used to assess certainty (or confidence) in the body of evidence for an outcome. | Not applicable |
| **RESULTS** | | |  |
| Study selection | 16a | Describe the results of the search and selection process, from the number of records identified in the search to the number of studies included in the review, ideally using a flow diagram. | 5, Figure:1 |
|  | 16b | Cite studies that might appear to meet the inclusion criteria, but which were excluded, and explain why they were excluded. | 5, Supplementary tables 6-11 |
| Study characteristics | 17 | Cite each included study and present its characteristics. | 6-8 |
| Risk of bias in studies | 18 | Present assessments of risk of bias for each included study. | 5,6 |
| Results of individual studies | 19 | For all outcomes, present, for each study: (a) summary statistics for each group (where appropriate) and (b) an effect estimate and its precision (e.g. confidence/credible interval), ideally using structured tables or plots. | 6-8 |
| Results of syntheses | 20a | For each synthesis, briefly summarise the characteristics and risk of bias among contributing studies. | 5-8 |
|  | 20b | Present results of all statistical syntheses conducted. If meta-analysis was done, present for each the summary estimate and its precision (e.g. confidence/credible interval) and measures of statistical heterogeneity. If comparing groups, describe the direction of the effect. | Table 3 and 4 |
|  | 20c | Present results of all investigations of possible causes of heterogeneity among study results. | Table 3 and 4 |
|  | 20d | Present results of all sensitivity analyses conducted to assess the robustness of the synthesised results. | Not applicable |
| Reporting biases/Quality assessment | 21 | Present assessments of risk of bias or Quality assessment of each study | Supplementary tables 6-11 |
| Certainty of evidence | 22 | Present assessments of certainty (or confidence) in the body of evidence for each outcome assessed. | Not applicable |
| **DISCUSSION** | | |  |
| Discussion | 23a | Provide a general interpretation of the results in the context of other evidence. | 8-10 |
|  | 23b | Discuss any limitations of the evidence included in the review. | 10 |
|  | 23c | Discuss any limitations of the review processes used. | 10 |
|  | 23d | Discuss implications of the results for practice, policy, and future research. | 9,10 |
| **OTHER INFORMATION** | | |  |
| Registration and protocol | 24a | Provide registration information for the review, including register name and registration number, or state that the review was not registered. | 10 |
|  | 24b | Indicate where the review protocol can be accessed, or state that a protocol was not prepared. | Not applicable |
|  | 24c | Describe and explain any amendments to information provided at registration or in the protocol. | Not applicable |
| Support | 25 | Describe sources of financial or non-financial support for the review, and the role of the funders or sponsors in the review. | 11 |
| Competing interests | 26 | Declare any competing interests of review authors. | 10,11 |
| Availability of data, code and other materials | 27 | Report which of the following are publicly available and where they can be found: template data collection forms; data extracted from included studies; data used for all analyses; analytic code; any other materials used in the review. | 10-26 |

*From:*  Page MJ, McKenzie JE, Bossuyt PM, Boutron I, Hoffmann TC, Mulrow CD, et al. The PRISMA 2020 statement: an updated guideline for reporting systematic reviews. BMJ 2021;372:n71. doi: 10.1136/bmj.n71 For more information, visit: <http://www.prisma-statement.org/>.

**SUPPLEMENTARY TABLE 2: PICO SEARCH STRATEGY**

| **PICO ELEMENTS** | **KEYWORDS** | **SEARCH TERMS** | **SEARCH STRATEGIES** |
| --- | --- | --- | --- |
| **P (Patient or Population)** | **Pregnant women** | Preeclampsia, hypertensive disorders of pregnancy, pregnancy complication | "preeclampsia" OR "pre-eclampsia" OR "pregnancy complication" OR "pregnancy-induced hypertension" OR "hypertensive disorders of pregnancy" OR "toxaemia" OR "toxemia" OR "maternal hypertension" OR "maternal syndrome" OR "pregnancy-specific disorder" OR "PE" OR "hypertension in pregnancy" OR "eclampsia" OR "HELPP syndrome" OR "haemolysis, elevated liver enzymes, low platelets" OR "EPH" OR "pregnancy toxemia" OR "edema-proteinuria-hypertension gestos" |
| **I (Intervention)** | **Genomic variations** linked to cardiovascular disease progression following preeclampsia | Genomic variation, genomic markers, genetic analyses, epigenetic modification, epigenetic analyses | "genetics" OR "genomics" OR "epigenomics" OR "DNA methylation" OR "epigenetics" OR "microRNA" OR "histone modification" OR "candidate gene studies" OR "association analysis" OR "linkage studies" OR "chromatin modification" OR "histones" OR "epigenetic modification" OR "epigenetic mechanism" OR "GWAS" OR "Genome-wide association studies" OR "Genome-wide association study" OR "GWLS" OR "Genome-wide linkage studies" OR "EWAS" OR "Epigenome-wide association studies" OR "Epigenome-wide DNA methylation studies" OR "Epigenome-wide association study" OR "non-coding RNAs" OR "translational regulation" OR "posttranslational regulation" OR "epigenetic changes" OR "transcriptional gene silencing" OR "posttranslational modification" OR "nucleosome remodelling" OR "nucleosome remodeling" OR "non-coding RNA regulation" OR "RNA editing" OR "gene-gene interactions" OR "gene-environmental interactions" OR "epistasis" OR "heritability" |
| **C (Comparison)** | Women with **a history of preeclampsia** Vs **normotensive pregnancy** monitored for the progression of later-life cardiovascular disease |  |  |
| **O (Outcome)** | Progression to **later-life cardiovascular disease** or **cardiovascular disease risk factors** | Cardiovascular disease, cardiovascular disease risk factors | "cardiovascular disease" OR "CVD" OR "Cardiovascular disease" OR "cardiovascular" OR "heart disease" OR "cardiometabolic risk" OR "stroke" OR "hypertension" OR "dyslipidemia" OR "dyslipidemia" OR "coronary heart disease" OR "cardiomyopathy" OR "congenital heart defects" OR "congenital heart disease" OR "heart arrhythmias" OR "atherosclerotic heart diseases" OR "rheumatic heart disease" OR "cardiac arrhythmias" OR "cerebrovascular disease" OR "coronary artery disease" OR "heart failure" OR "heart valve disease" OR "ischaemic heart disease" OR "ischemic heart disease" OR "atherosclerosis" OR "heart attack" OR "inflammatory heart disease" OR "heart disorder" OR "myocardial" OR "ventricular arrhythmia" OR "cardiac arrest" OR "abnormal heart rhythms" OR "hypertensive heart disease" OR "carditis" OR "peripheral artery disease" OR "thromboembolic disease" OR "myocardial infarction" OR "venous thrombosis" OR "acute coronary syndrome" OR "cardiac failure" OR "acute coronary syndrome" OR "left ventricular systolic dysfunction" OR "venous thromboembolism" OR "cerebrovascular accident" OR "deep vein thrombosis" |

**SUPPLEMENTARY TABLE 3: SEARCH STRATEGY (27/08/2021)**

| **SCOPUS**  #1: TITLE ("preeclampsia" OR "pre-eclampsia" OR "pregnancy complication" OR "pregnancy-induced hypertension" OR "hypertensive disorders of pregnancy" OR "toxaemia" OR "toxemia" OR "maternal hypertension" OR "maternal syndrome" OR "pregnancy-specific disorder" OR "PE" OR "hypertension in pregnancy" OR "eclampsia" OR "HELPP syndrome" OR "haemolysis, elevated liver enzymes, low platelets" OR "EPH" OR "pregnancy toxemia" OR "edema-proteinuria-hypertension gestos") OR ABS ("preeclampsia" OR "pre-eclampsia" OR "pregnancy complication" OR "pregnancy-induced hypertension" OR "hypertensive disorders of pregnancy" OR "toxaemia" OR "toxemia" OR "maternal hypertension" OR "maternal syndrome" OR "pregnancy-specific disorder" OR "PE" OR "hypertension in pregnancy" OR "eclampsia" OR "HELPP syndrome" OR "haemolysis, elevated liver enzymes, low platelets" OR "EPH" OR "pregnancy toxemia" OR "edema-proteinuria-hypertension gestos"): 155,525 results  #2: TITLE ("cardiovascular disease" OR "CVD" OR "Cardiovascular disease" OR "cardiovascular" OR "heart disease" OR "cardiometabolic risk" OR "stroke" OR "hypertension" OR "dyslipidemia" OR "dyslipidemia" OR "coronary heart disease" OR "cardiomyopathy" OR "congenital heart defects" OR "congenital heart disease" OR "heart arrhythmias" OR "atherosclerotic heart diseases" OR "rheumatic heart disease" OR "cardiac arrhythmias" OR "cerebrovascular disease" OR "coronary artery disease" OR "heart failure" OR "heart valve disease" OR "ischaemic heart disease" OR "ischemic heart disease" OR "atherosclerosis" OR "heart attack" OR "inflammatory heart disease" OR "heart disorder" OR "myocardial" OR "ventricular arrhythmia" OR "cardiac arrest" OR "abnormal heart rhythms" OR "hypertensive heart disease" OR "carditis" OR "peripheral artery disease" OR "thromboembolic disease" OR "myocardial infarction" OR "venous thrombosis" OR "acute coronary syndrome" OR "cardiac failure" OR "acute coronary syndrome" OR "left ventricular systolic dysfunction" OR "venous thromboembolism" OR "cerebrovascular accident" OR "deep vein thrombosis" ) OR ABS ( "cardiovascular disease" OR "CVD" OR "Cardiovascular disease" OR "cardiovascular" OR "heart disease" OR "cardiometabolic risk" OR "stroke" OR "hypertension" OR "dyslipidemia" OR "dyslipidemia" OR "coronary heart disease" OR "cardiomyopathy" OR "congenital heart defects" OR "congenital heart disease" OR "heart arrhythmias" OR "atherosclerotic heart diseases" OR "rheumatic heart disease" OR "cardiac arrhythmias" OR "cerebrovascular disease" OR "coronary artery disease" OR "heart failure" OR "heart valve disease" OR "ischaemic heart disease" OR "ischemic heart disease" OR "atherosclerosis" OR "heart attack" OR "inflammatory heart disease" OR "heart disorder" OR "myocardial" OR "ventricular arrhythmia" OR "cardiac arrest" OR "abnormal heart rhythms" OR "hypertensive heart disease" OR "carditis" OR "peripheral artery disease" OR "thromboembolic disease" OR "myocardial infarction" OR "venous thrombosis" OR "acute coronary syndrome" OR "cardiac failure" OR "acute coronary syndrome" OR "left ventricular systolic dysfunction" OR "venous thromboembolism" OR "cerebrovascular accident" OR "deep vein thrombosis"): 2,178,704 results  #3: ALL ( "genetics" OR "genomics" OR "epigenomics" OR "DNA methylation" OR "epigenetics" OR "microRNA" OR "histone modification" OR "candidate gene studies" OR "association analysis" OR "linkage studies" OR "chromatin modification" OR "histones" OR "epigenetic modification" OR "epigenetic mechanism" OR "GWAS" OR "Genome-wide association studies" OR "Genome-wide association study" OR "GWLS" OR "Genome-wide linkage studies" OR "EWAS" OR "Epigenome-wide association studies" OR "Epigenome-wide DNA methylation studies" OR "Epigenome-wide association study" OR "non-coding RNAs" OR "translational regulation" OR "posttranslational regulation" OR "epigenetic changes" OR "transcriptional gene silencing" OR "posttranslational modification" OR "nucleosome remodelling" OR "nucleosome remodeling" OR "non-coding RNA regulation" OR "RNA editing" OR "gene-gene interactions" OR "gene-environmental interactions" OR "epistasis" OR "heritability"): 6,085,974 results  #4: ALL ( "shared" OR "associated" OR "related" OR "colocalization" OR "colocalisation" OR "relation" OR "correlated" OR "association" OR "correlation" OR "link" OR "connected" OR "connecting" OR "involved" OR "involving" OR "combined" OR "combining" OR "later-life" OR "later life" OR "later in life" OR "later risk" OR "future risk" OR "long term" OR "later-risk") : 42,179,901 results  #1 AND #2 AND #3 AND #4 AND ( LIMIT-TO ( PUBYEAR , 2021 ) OR LIMIT-TO ( PUBYEAR , 2020 ) OR LIMIT-TO ( PUBYEAR , 2019 ) OR LIMIT-TO ( PUBYEAR , 2018 ) OR LIMIT-TO ( PUBYEAR , 2017 ) OR LIMIT-TO ( PUBYEAR , 2016 ) OR LIMIT-TO ( PUBYEAR , 2015 ) OR LIMIT-TO ( PUBYEAR , 2014 ) OR LIMIT-TO ( PUBYEAR , 2013 ) OR LIMIT-TO ( PUBYEAR , 2012 ) OR LIMIT-TO ( PUBYEAR , 2011 ) OR LIMIT-TO ( PUBYEAR , 2010 ) OR LIMIT-TO ( PUBYEAR , 2009 ) OR LIMIT-TO ( PUBYEAR , 2008 ) OR LIMIT-TO ( PUBYEAR , 2007 ) OR LIMIT-TO ( PUBYEAR , 2006 ) OR LIMIT-TO ( PUBYEAR , 2005 ) OR LIMIT-TO ( PUBYEAR , 2004 ) OR LIMIT-TO ( PUBYEAR , 2003 ) OR LIMIT-TO ( PUBYEAR , 2002 ) OR LIMIT-TO ( PUBYEAR , 2001 ) OR LIMIT-TO ( PUBYEAR , 2000 ) OR LIMIT-TO ( PUBYEAR , 1999 ) OR LIMIT-TO ( PUBYEAR , 1998 ) OR LIMIT-TO ( PUBYEAR , 1997 ) OR LIMIT-TO ( PUBYEAR , 1996 ) OR LIMIT-TO ( PUBYEAR , 1995 ) OR LIMIT-TO ( PUBYEAR , 1994 ) OR LIMIT-TO ( PUBYEAR , 1993 ) OR LIMIT-TO ( PUBYEAR , 1992 ) OR LIMIT-TO ( PUBYEAR , 1991 ) OR LIMIT-TO ( PUBYEAR , 1990 ) OR LIMIT-TO ( PUBYEAR , 1989 ) OR LIMIT-TO ( PUBYEAR , 1988 ) OR LIMIT-TO ( PUBYEAR , 1986 ) OR LIMIT-TO ( PUBYEAR , 1985 ) OR LIMIT-TO ( PUBYEAR , 1984 ) OR LIMIT-TO ( PUBYEAR , 1983 ) OR LIMIT-TO ( PUBYEAR , 1982 ) OR LIMIT-TO ( PUBYEAR , 1981 ) ) AND ( LIMIT-TO ( DOCTYPE , "ar" ) ) AND ( LIMIT-TO ( LANGUAGE , "English" ) ) : 2646 RESULTS |
| --- |
| **WEB OF SCIENCE**  #1 TI=(‘preeclampsia’ OR ‘pre-eclampsia’ OR ‘Preeclampsia’ OR ‘Pre-eclampsia’ OR ‘pregnancy complication’ OR ‘pregnancy-induced hypertension’ OR ‘hypertensive disorders of pregnancy’ OR ‘toxaemia’ OR ‘toxemia’ OR ‘maternal hypertension’ OR ‘maternal syndrome’ OR ‘pregnancy-specific disorder’ OR ‘PE’ OR ‘hypertension in pregnancy’ OR ‘eclampsia’ OR ‘HELPP syndrome’ OR ‘haemolysis, elevated liver enzymes, low platelets’ OR ‘EPH’ OR ‘pregnancy toxemia’ OR ‘edema-proteinuria-hypertension gestos’) OR AB=(‘preeclampsia’ OR ‘pre-eclampsia’ OR ‘Preeclampsia’ OR ‘Pre-eclampsia’ OR ‘pregnancy complication’ OR ‘pregnancy-induced hypertension’ OR ‘hypertensive disorders of pregnancy’ OR ‘toxaemia’ OR ‘toxemia’ OR ‘maternal hypertension’ OR ‘maternal syndrome’ OR ‘pregnancy-specific disorder’ OR ‘PE’ OR ‘hypertension in pregnancy’ OR ‘eclampsia’ OR ‘HELPP syndrome’ OR ‘haemolysis, elevated liver enzymes, low platelets’ OR ‘EPH’ OR ‘pregnancy toxemia’ OR ‘edema-proteinuria-hypertension gestos’) and 2021 or 2020 or 2019 or 2018 or 2017 or 2016 or 2014 or 2015 or 2013 or 2012 or 2010 or 2009 or 2008 or 2005 or 2006 or 2007 or 2011 or 2004 or 2003 or 2001 or 2000 or 2002 or 1999 or 1997 or 1996 or 1998 or 1995 or 1994 or 1993 or 1992 or 1991 or 1990 or 1989 or 1988 or 1987 or 1986 or 1985 or 1984 or 1983 or 1982 or 1981 or 1980 (Publication Years) and Articles (Document Types) and English (Languages): 71,483 results  #2 TI=(‘cardiovascular disease’ OR ‘CVD’ OR ‘Cardiovascular disease’ OR ‘cardiovascular’ OR ‘heart disease’ OR ‘cardiometabolic risk’ OR ‘stroke’ OR ‘hypertension’ OR ‘dyslipidemia’ OR ‘dyslipidaemia’ OR ‘coronary heart disease’ OR ‘cardiomyopathy’ OR ‘congenital heart defects’ OR ‘congenital heart disease’ OR ‘heart arrhythmias’ OR ‘atherosclerotic heart diseases’ OR ‘rheumatic heart disease’ OR ‘cardiac arrhythmias’ OR ‘cerebrovascular disease’ OR ‘coronary artery disease’ OR ‘heart failure’ OR ‘heart valve disease’ OR ‘ischaemic heart disease’ OR ‘ischemic heart disease’ OR ‘atherosclerosis’ OR ‘heart attack’ OR ‘inflammatory heart disease’ OR ‘heart disorder’ OR ‘myocardial’ OR ‘ventricular arrhythmia’ OR ‘cardiac arrest’ OR ‘abnormal heart rhythms’ OR ‘hypertensive heart disease’ OR ‘carditis’ OR ‘peripheral artery disease’ OR ‘thromboembolic disease’ OR ‘myocardial infarction’ OR ‘venous thrombosis’ OR ‘acute coronary syndrome’ OR ‘cardiac failure’ OR ‘acute coronary syndrome’ OR ‘left ventricular systolic dysfunction’ OR ‘venous thromboembolism’ OR ‘cerebrovascular accident’ OR ‘deep vein thrombosis’) OR AB=(‘cardiovascular disease’ OR ‘CVD’ OR ‘Cardiovascular disease’ OR ‘cardiovascular’ OR ‘heart disease’ OR ‘cardiometabolic risk’ OR ‘stroke’ OR ‘hypertension’ OR ‘dyslipidemia’ OR ‘dyslipidaemia’ OR ‘coronary heart disease’ OR ‘cardiomyopathy’ OR ‘congenital heart defects’ OR ‘congenital heart disease’ OR ‘heart arrhythmias’ OR ‘atherosclerotic heart diseases’ OR ‘rheumatic heart disease’ OR ‘cardiac arrhythmias’ OR ‘cerebrovascular disease’ OR ‘coronary artery disease’ OR ‘heart failure’ OR ‘heart valve disease’ OR ‘ischaemic heart disease’ OR ‘ischemic heart disease’ OR ‘atherosclerosis’ OR ‘heart attack’ OR ‘inflammatory heart disease’ OR ‘heart disorder’ OR ‘myocardial’ OR ‘ventricular arrhythmia’ OR ‘cardiac arrest’ OR ‘abnormal heart rhythms’ OR ‘hypertensive heart disease’ OR ‘carditis’ OR ‘peripheral artery disease’ OR ‘thromboembolic disease’ OR ‘myocardial infarction’ OR ‘venous thrombosis’ OR ‘acute coronary syndrome’ OR ‘cardiac failure’ OR ‘acute coronary syndrome’ OR ‘left ventricular systolic dysfunction’ OR ‘venous thromboembolism’ OR ‘cerebrovascular accident’ OR ‘deep vein thrombosis’) and 2021 or 2019 or 2020 or 2018 or 2017 or 2016 or 2015 or 2014 or 2013 or 2012 or 2011 or 2010 or 2009 or 2008 or 2006 or 2007 or 2005 or 2004 or 2003 or 2000 or 2001 or 2002 or 1998 or 1999 or 1997 or 1995 or 1996 or 1994 or 1992 or 1993 or 1991 or 1990 or 1989 or 1988 or 1987 or 1985 or 1986 or 1980 or 1981 or 1982 or 1983 or 1984 (Publication Years) and Articles (Document Types) and English (Languages): 1,155,806 results  #3 ALL=(‘genetic’ OR ‘candidate gene studies’ OR ‘association analysis’ OR ‘linkage studies’ OR ‘GWAS’ OR ‘Genome-wide association studies’ OR ‘Genome-wide association study’ OR ‘GWLS’ OR ‘Genome-wide linkage studies’ OR ‘gene-gene interactions’ OR ‘gene-environmental interactions’ OR ‘epistasis’ OR ‘heritability’ ) and 2022 or 2021 or 2020 or 2019 or 2018 or 2017 or 2016 or 2015 or 2014 or 2013 or 2012 or 2011 or 2010 or 2009 or 2008 or 2007 or 2006 or 2005 or 2004 or 2003 or 2002 or 2001 or 2000 or 1999 or 1998 or 1997 or 1996 or 1995 or 1994 or 1993 or 1992 or 1980 or 1981 or 1982 or 1983 or 1984 or 1985 or 1986 or 1987 or 1988 or 1989 or 1990 or 1991 (Publication Years) and English (Languages) and Articles (Document Types) 1,869,978 results  #4 ALL=(‘DNA methylation’ OR ‘epigenetics’ OR ‘microRNA’ OR ‘histone modification’ OR ‘chromatin modification’ OR ‘histones’ OR ‘epigenetic modification’ OR ‘epigenetic mechanism’ OR ‘EWAS’ OR ‘Epigenome-wide association studies’ OR ‘Epigenome-wide DNA methylation studies’ OR ‘Epigenome-wide association study’ OR ‘non-coding RNAs’ OR ‘translational regulation’ OR ‘posttranslational regulation’ OR ‘epigenetic changes’ OR ‘transcriptional gene silencing’ OR ‘posttranslational modification’ OR ‘nucleosome remodelling’ OR ‘nucleosome remodeling’ OR ‘non-coding RNA regulation’ OR ‘RNA editing’ ) and 2022 or 2021 or 2020 or 2019 or 2018 or 2017 or 2016 or 2015 or 2014 or 2013 or 2012 or 2011 or 2010 or 2009 or 2008 or 1980 or 1981 or 1982 or 1983 or 1984 or 1985 or 1986 or 1987 or 1988 or 1989 or 1990 or 1991 or 1992 or 1993 or 1994 or 1995 or 1996 or 1997 or 1998 or 1999 or 2000 or 2001 or 2002 or 2003 or 2007 or 2006 or 2005 or 2004 (Publication Years) and Articles (Document Types) and English (Languages): 307,783 results  #5 ALL=(‘shared’ OR ‘associated’ OR ‘related’ OR ‘colocalisation’ OR ‘colocalisation’ OR ‘relation’ OR ‘correlated’ OR ‘association’ OR ‘correlation’ OR ‘link’ OR ‘connected’ OR ‘connecting’ OR ‘involved’ OR ‘involving’ OR ‘combined’ OR ‘combining’ OR ‘later-life’ OR ‘later life’ OR ‘later in life’ OR ‘later risk’ OR ‘future risk’ OR ‘long term’ OR ‘later-risk’ ) and 2022 or 2021 or 2020 or 2019 or 2018 or 2017 or 2016 or 2014 or 2015 or 2013 or 2012 or 2011 or 2010 or 2009 or 2008 or 2007 or 2006 or 2005 or 2004 or 2003 or 2002 or 2001 or 2000 or 1999 or 1998 or 1997 or 1996 or 1994 or 1995 or 1993 or 1992 or 1991 or 1990 or 1989 or 1988 or 1987 or 1986 or 1985 or 1984 or 1983 or 1982 or 1981 or 1980 (Publication Years) and Articles (Document Types) and English (Languages): 14,734,868 results  #1 AND #2 AND (#3 OR #4) AND #5: 2851 RESULTS |
| **PUBMED**  #1: ‘preeclampsia’ OR ‘pre-eclampsia’ OR ‘Preeclampsia’ OR ‘Pre-eclampsia’ OR ‘pregnancy complication’ OR ‘pregnancy-induced hypertension’ OR ‘hypertensive disorders of pregnancy’ OR ‘toxaemia’ OR ‘toxemia’ OR ‘maternal hypertension’ OR ‘maternal syndrome’ OR ‘pregnancy-specific disorder’ OR ‘PE’ OR ‘hypertension in pregnancy’ OR ‘eclampsia’ OR ‘HELPP syndrome’ OR ‘haemolysis, elevated liver enzymes, low platelets’ OR ‘EPH’ OR ‘pregnancy toxemia’ OR ‘edema-proteinuria-hypertension gestos’ (Title/ Abstract): 51,205 results  #2: ‘cardiovascular disease’ OR ‘CVD’ OR ‘Cardiovascular disease’ OR ‘cardiovascular’ OR ‘heart disease’ OR ‘cardiometabolic risk’ OR ‘stroke’ OR ‘hypertension’ OR ‘dyslipidemia’ OR ‘dyslipidaemia’ OR ‘coronary heart disease’ OR ‘cardiomyopathy’ OR ‘congenital heart defects’ OR ‘congenital heart disease’ OR ‘heart arrhythmias’ OR ‘atherosclerotic heart diseases’ OR ‘rheumatic heart disease’ OR ‘cardiac arrhythmias’ OR ‘cerebrovascular disease’ OR ‘coronary artery disease’ OR ‘heart failure’ OR ‘heart valve disease’ OR ‘ischaemic heart disease’ OR ‘ischemic heart disease’ OR ‘atherosclerosis’ OR ‘heart attack’ OR ‘inflammatory heart disease’ OR ‘heart disorder’ OR ‘myocardial’ OR ‘ventricular arrhythmia’ OR ‘cardiac arrest’ OR ‘abnormal heart rhythms’ OR ‘hypertensive heart disease’ OR ‘carditis’ OR ‘peripheral artery disease’ OR ‘thromboembolic disease’ OR ‘myocardial infarction’ OR ‘venous thrombosis’ OR ‘acute coronary syndrome’ OR ‘cardiac failure’ OR ‘acute coronary syndrome’ OR ‘left ventricular systolic dysfunction’ OR ‘venous thromboembolism’ OR ‘cerebrovascular accident’ OR ‘deep vein thrombosis’ (Title/abstract): 1,039,905 results  #3: ‘genetic’ OR ‘genomics’ OR ‘epigenomics’ OR ‘DNA methylation’ OR ‘epigenetics’ OR ‘microRNA’ OR ‘histone modification’ OR ‘candidate gene studies’ OR ‘association analysis’ OR ‘linkage studies’ OR ‘chromatin modification’ OR ‘histones’ OR ‘epigenetic modification’ OR ‘epigenetic mechanism’ OR ‘GWAS’ OR ‘Genome-wide association studies’ OR ‘Genome-wide association study’ OR ‘GWLS’ OR ‘Genome-wide linkage studies’ OR ‘EWAS’ OR ‘Epigenome-wide association studies’ OR ‘Epigenome-wide DNA methylation studies’ OR ‘Epigenome-wide association study’ OR ‘non-coding RNAs’ OR ‘translational regulation’ OR ‘posttranslational regulation’ OR ‘epigenetic changes’ OR ‘transcriptional gene silencing’ OR ‘posttranslational modification’ OR ‘nucleosome remodelling’ OR ‘nucleosome remodeling’ OR ‘non-coding RNA regulation’ OR ‘RNA editing’ OR ‘gene-gene interactions’ OR ‘gene-environmental interactions’ OR ‘epistasis’ OR ‘heritability’(All fields):3,303,640 results  #4: ‘shared’ OR ‘associated’ OR ‘related’ OR ‘colocalization’ OR ‘colocalisation’ OR ‘relation’ OR ‘correlated’ OR ‘association’ OR ‘correlation’ OR ‘link’ OR ‘connected’ OR ‘connecting’ OR ‘involved’ OR ‘involving’ OR ‘combined’ OR ‘combining’ OR ‘later-life’ OR ‘later life’ OR ‘later in life’ OR ‘later risk’ OR ‘future risk’ OR ‘long term’ OR ‘later-risk’ (All fields): 7,110,736 results  #1 AND #2 AND #3 AND #4: 4774 RESULTS  Filters applied: Journal Article, Humans, English, Year: post-1980s |
| **EMBASE via OVID**  Embase <1974 to 2021 August 25>  1 preeclampsia.ab,ti. 37231  2 "pre-eclampsia".ab,ti. 15889  3 Preeclampsia.ab,ti. 37231  4 "Pre-eclampsia".ab,ti. 15889  5 "pregnancy complication".ab,ti. 1174  6 "pregnancy-induced hypertension".ab,ti. 5409  7 "hypertensive disorders of pregnancy".ab,ti. 4090  8 toxaemia.ab,ti. 711  9 toxemia.ab,ti. 2588  10 "maternal hypertension".ab,ti. 1233  11 "maternal syndrome".ab,ti. 188  12 "pregnancy-specific disorder".ab,ti. 190  13 "hypertension in pregnancy".ab,ti. 2383  14 eclampsia.ab,ti. 21963  15 "HELPP syndrome".ab,ti. 29  16 "haemolysis, elevated liver enzymes, low platelets".ab,ti. 66  17 "pregnancy toxemia".ab,ti. 323  18 "edema-proteinuria-hypertension gestos".ab,ti. 0  19 EPH.ab,ti. 3710  20 PE.ab,ti. 69173  21 1 or 2 or 3 or 4 or 5 or 6 or 7 or 8 or 9 or 10 or 11 or 12 or 13 or 14 or 15 or 16 or 17 or 18 or 19 or 20 130219  22 limit 21 to (human and english language and yr="1980 - 2021" and article) 50062  23 "cardiovascular disease".ab,ti. 201447  24 "Cardiovascular disease".ab,ti. 201447  25 cardiovascular.ab,ti. 698794  26 "heart disease".ab,ti. 227523  27 "cardiometabolic risk".ab,ti. 9047  28 stroke.ab,ti. 417378  29 hypertension.ab,ti. 612568  30 dyslipidemia.ab,ti. 53636  31 dyslipidaemia.ab,ti. 9483  32 "coronary heart disease".ab,ti. 68842  33 cardiomyopathy.ab,ti. 115203  34 "congenital heart defects".ab,ti. 9301  35 "congenital heart disease".ab,ti. 39638  36 "heart arrhythmias".ab,ti. 288  37 "atherosclerotic heart diseases".ab,ti. 34  38 "rheumatic heart disease".ab,ti. 5790  39 "cardiac arrhythmias".ab,ti. 14843  40 "cerebrovascular disease".ab,ti. 24369  41 "coronary artery disease".ab,ti. 139474  42 "heart failure".ab,ti. 299418  43 "heart valve disease".ab,ti. 1157  44 "ischaemic heart disease".ab,ti. 12230  45 "ischemic heart disease".ab,ti. 38812  46 "atherosclerosis".ab,ti. 163092  47 "heart attack".ab,ti. 6811  48 "inflammatory heart disease".ab,ti. 269  49 "heart disorder".ab,ti. 216  50 "myocardial".ab,ti. 496202  51 "ventricular arrhythmia".ab,ti. 9005  52 "cardiac arrest".ab,ti. 58178  53 "abnormal heart rhythms".ab,ti. 73  54 "hypertensive heart disease".ab,ti. 2502  55 carditis.ab,ti. 2075  56 "peripheral artery disease".ab,ti. 9730  57 "thromboembolic disease".ab,ti. 5949  58 "myocardial infarction".ab,ti. 278845  59 "venous thrombosis".ab,ti. 39590  60 "acute coronary syndrome".ab,ti. 44482  61 "cardiac failure".ab,ti. 17481  62 "acute coronary syndrome".ab,ti. 44482  63 "left ventricular systolic dysfunction".ab,ti. 5625  64 "venous thromboembolism".ab,ti. 36751  65 "cerebrovascular accident".ab,ti. 7472  66 "deep vein thrombosis".ab,ti. 27141  67 23 or 24 or 25 or 26 or 27 or 28 or 29 or 30 or 31 or 32 or 33 or 34 or 35 or 36 or 37 or 38 or 39 or 40 or 41 or 42 or 43 or 44 or 45 or 46 or 47 or 48 or 49 or 50 or 51 or 52 or 53 or 54 or 55 or 56 or 57 or 58 or 59 or 60 or 61 or 62 or 63 or 64 or 65 or 66 2500833  68 limit 67 to (human and english language and yr="1980 - 2021" and article) 943627  69 genetic*.af. 3405477  70 genomic*.af. 668980  71 epigenomic*.af. 11700  72 "DNA methylation".af. 98366  73 epigenetic*.af. 166142  74 microRNA.ab,ti. 77377  75 "histone modification".af. 19466  76 "candidate gene stud*".af. 1787  77 "association analysis".af. 11029  78 "linkage stud*".af. 6954  79 "chromatin modification".af. 2249  80 histones.af. 21303  81 "epigenetic modification".af. 5891  82 "epigenetic mechanism".af. 3570  83 GWAS.af. 27769  84 "Genome-wide association studies".af. 25587  85 "Genome-wide association study".af. 40942  86 GWLS.af. 52  87 "Genome-wide linkage study".af. 193  88 "Genome-wide linkage studies".af. 220  89 GWLS.af. 52  90 EWAS.af. 851  91 "Epigenome-wide association studies".af. 483  92 "Epigenome-wide DNA methylation stud*".af. 11  93 "Epigenome-wide association study".af. 690  94 "non-coding RNAs".af. 24650  95 "translational regulation".af. 5081  96 "posttranslational regulation".af. 915  97 "epigenetic changes".af. 11662  98 "transcriptional gene silencing".af. 1826  99 "posttranslational modification".af. 7841  100 "nucleosome remodelling".af. 214  101 "nucleosome remodeling".af. 1186  102 "non-coding RNA regulation".af. 64  103 "gene-gene interactions".af. 2222  104 "gene-environmental interactions".af. 335  105 epistasis.af. 9159  106 heritability.af. 29605  107 69 or 70 or 71 or 72 or 73 or 74 or 75 or 76 or 77 or 78 or 79 or 80 or 81 or 82 or 83 or 84 or 85 or 86 or 87 or 88 or 89 or 90 or 91 or 92 or 93 or 94 or 95 or 96 or 97 or 98 or 99 or 100 or 101 or 102 or 103 or 104 or 105 or 106 3834222  108 limit 107 to (human and english language and yr="1980 - 2021" and article) 1262279  109 shared.af. 204476  110 associated.af. 5555396  111 related.af. 3675515  112 colocalization.af. 18146  113 colocalisation.af. 913  114 relation.af. 1108025  115 correlated.af. 1116678  116 association.af. 4180241  117 correlation.af. 1477550  118 link.af. 528293  119 connected.af. 134652  120 connecting.af. 40459  121 involved.af. 1674732  122 involving.af. 564838  123 combined.af. 1116290  124 combining.af. 228545  125 "later-life".af. 15688  126 "later life".af. 15688  127 "later in life".af. 16851  128 "later risk".af. 624  129 "future risk".af. 5111  130 "long term".af. 1287195  131 "later-risk".af. 624  132 "future risk".af. 5111  133 "long term".af. 1287195  134 "later-risk".af. 624  135 109 or 110 or 111 or 112 or 113 or 114 or 115 or 116 or 117 or 118 or 119 or 120 or 121 or 122 or 123 or 124 or 125 or 126 or 127 or 128 or 129 or 130 or 131 or 132 or 133 or 134 15200786  136 limit 135 to (human and english language and yr="1980 - 2021" and article) 6115539  137 22 and 68 and 108 and 136: 922 RESULTS |
| **Summary:**  Scopus: 2646 RESULTS  Web of Science: 2851 RESULTS  PubMed: 4774 RESULTS  EMBASE via OVID: 922 RESULTS  Total: 11,193 RESULTS |

**SUPPLEMENTARY TABLE 4: UPDATED SEARCH (02/02/2023)**

| **SCOPUS**  #1: TITLE ( "preeclampsia" OR "pre-eclampsia" OR "pregnancy complication" OR "pregnancy-induced hypertension" OR "hypertensive disorders of pregnancy" OR "toxaemia" OR "toxemia" OR "maternal hypertension" OR "maternal syndrome" OR "pregnancy-specific disorder" OR "pe" OR "hypertension in pregnancy" OR "eclampsia" OR "helpp syndrome" OR "haemolysis, elevated liver enzymes, low platelets" OR "eph" OR "pregnancy toxemia" OR "edema-proteinuria-hypertension gestos" ) OR ABS ( "preeclampsia" OR "pre-eclampsia" OR "pregnancy complication" OR "pregnancy-induced hypertension" OR "hypertensive disorders of pregnancy" OR "toxaemia" OR "toxemia" OR "maternal hypertension" OR "maternal syndrome" OR "pregnancy-specific disorder" OR "pe" OR "hypertension in pregnancy" OR "eclampsia" OR "helpp syndrome" OR "haemolysis, elevated liver enzymes, low platelets" OR "eph" OR "pregnancy toxemia" OR "edema-proteinuria-hypertension gestos" ) AND ( LIMIT-TO ( PUBYEAR , 2023 ) OR LIMIT-TO ( PUBYEAR , 2022 ) OR LIMIT-TO ( PUBYEAR , 2021 ) ) AND ( LIMIT-TO ( DOCTYPE , "ar" ) ) AND ( LIMIT-TO ( LANGUAGE , "English" ) ) AND ( LIMIT-TO ( SRCTYPE , "j" ) : 18,424 results  #2: TITLE ( "cardiovascular disease" OR "CVD" OR "Cardiovascular disease" OR "cardiovascular" OR "heart disease" OR "cardiometabolic risk" OR "stroke" OR "hypertension" OR "dyslipidemia" OR "dyslipidemia" OR "coronary heart disease" OR "cardiomyopathy" OR "congenital heart defects" OR "congenital heart disease" OR "heart arrhythmias" OR "atherosclerotic heart diseases" OR "rheumatic heart disease" OR "cardiac arrhythmias" OR "cerebrovascular disease" OR "coronary artery disease" OR "heart failure" OR "heart valve disease" OR "ischaemic heart disease" OR "ischemic heart disease" OR "atherosclerosis" OR "heart attack" OR "inflammatory heart disease" OR "heart disorder" OR "myocardial" OR "ventricular arrhythmia" OR "cardiac arrest" OR "abnormal heart rhythms" OR "hypertensive heart disease" OR "carditis" OR "peripheral artery disease" OR "thromboembolic disease" OR "myocardial infarction" OR "venous thrombosis" OR "acute coronary syndrome" OR "cardiac failure" OR "acute coronary syndrome" OR "left ventricular systolic dysfunction" OR "venous thromboembolism" OR "cerebrovascular accident" OR "deep vein thrombosis" ) OR ABS ( "cardiovascular disease" OR "CVD" OR "Cardiovascular disease" OR "cardiovascular" OR "heart disease" OR "cardiometabolic risk" OR "stroke" OR "hypertension" OR "dyslipidemia" OR "dyslipidemia" OR "coronary heart disease" OR "cardiomyopathy" OR "congenital heart defects" OR "congenital heart disease" OR "heart arrhythmias" OR "atherosclerotic heart diseases" OR "rheumatic heart disease" OR "cardiac arrhythmias" OR "cerebrovascular disease" OR "coronary artery disease" OR "heart failure" OR "heart valve disease" OR "ischaemic heart disease" OR "ischemic heart disease" OR "atherosclerosis" OR "heart attack" OR "inflammatory heart disease" OR "heart disorder" OR "myocardial" OR "ventricular arrhythmia" OR "cardiac arrest" OR "abnormal heart rhythms" OR "hypertensive heart disease" OR "carditis" OR "peripheral artery disease" OR "thromboembolic disease" OR "myocardial infarction" OR "venous thrombosis" OR "acute coronary syndrome" OR "cardiac failure" OR "acute coronary syndrome" OR "left ventricular systolic dysfunction" OR "venous thromboembolism" OR "cerebrovascular accident" OR "deep vein thrombosis" ) AND ( LIMIT-TO ( PUBYEAR , 2023 ) OR LIMIT-TO ( PUBYEAR , 2022 ) OR LIMIT-TO ( PUBYEAR , 2021 ) ) AND ( LIMIT-TO ( DOCTYPE , "ar" ) ) AND ( LIMIT-TO ( LANGUAGE , "English" ) ) AND ( LIMIT-TO ( SRCTYPE , "j" ) ): 186,782 results  #3: ALL ( "genetics" OR "genomics" OR "epigenomics" OR "DNA methylation" OR "epigenetics" OR "microRNA" OR "histone modification" OR "candidate gene studies" OR "association analysis" OR "linkage studies" OR "chromatin modification" OR "histones" OR "epigenetic modification" OR "epigenetic mechanism" OR "GWAS" OR "Genome-wide association studies" OR "Genome-wide association study" OR "GWLS" OR "Genome-wide linkage studies" OR "EWAS" OR "Epigenome-wide association studies" OR "Epigenome-wide DNA methylation studies" OR "Epigenome-wide association study" OR "non-coding RNAs" OR "translational regulation" OR "posttranslational regulation" OR "epigenetic changes" OR "transcriptional gene silencing" OR "posttranslational modification" OR "nucleosome remodelling" OR "nucleosome remodeling" OR "non-coding RNA regulation" OR "RNA editing" OR "gene-gene interactions" OR "gene-environmental interactions" OR "epistasis" OR "heritability" ) AND ( LIMIT-TO ( PUBYEAR , 2023 ) OR LIMIT-TO ( PUBYEAR , 2022 ) OR LIMIT-TO ( PUBYEAR , 2021 ) ) AND ( LIMIT-TO ( DOCTYPE , "ar" ) ) AND ( LIMIT-TO ( LANGUAGE , "English" ) ) AND ( LIMIT-TO ( SRCTYPE , "j" ) ): 719,966 results  #4: ALL ( "shared" OR "associated" OR "related" OR "colocalization" OR "colocalisation" OR "relation" OR "correlated" OR "association" OR "correlation" OR "link" OR "connected" OR "connecting" OR "involved" OR "involving" OR "combined" OR "combining" OR "later-life" OR "later life" OR "later in life" OR "later risk" OR "future risk" OR "long term" OR "later-risk" ) AND ( LIMIT-TO ( PUBYEAR , 2023 ) OR LIMIT-TO ( PUBYEAR , 2022 ) OR LIMIT-TO ( PUBYEAR , 2021 ) ) AND ( LIMIT-TO ( DOCTYPE , "ar" ) ) AND ( LIMIT-TO ( LANGUAGE , "English" ) ) AND ( LIMIT-TO ( SRCTYPE , "j" ) ): 4,442,720 results  #1 AND #2 AND #3 AND #4: 663 RESULTS |
| --- |
| **WEB OF SCIENCE**  #1: (TI=(‘preeclampsia’ OR ‘pre-eclampsia’ OR ‘Preeclampsia’ OR ‘Pre-eclampsia’ OR ‘pregnancy complication’ OR ‘pregnancy-induced hypertension’ OR ‘hypertensive disorders of pregnancy’ OR ‘toxaemia’ OR ‘toxemia’ OR ‘maternal hypertension’ OR ‘maternal syndrome’ OR ‘pregnancy-specific disorder’ OR ‘PE’ OR ‘hypertension in pregnancy’ OR ‘eclampsia’ OR ‘HELPP syndrome’ OR ‘haemolysis, elevated liver enzymes, low platelets’ OR ‘EPH’ OR ‘pregnancy toxemia’ OR ‘edema-proteinuria-hypertension gestos’)) OR AB=(‘preeclampsia’ OR ‘pre-eclampsia’ OR ‘Preeclampsia’ OR ‘Pre-eclampsia’ OR ‘pregnancy complication’ OR ‘pregnancy-induced hypertension’ OR ‘hypertensive disorders of pregnancy’ OR ‘toxaemia’ OR ‘toxemia’ OR ‘maternal hypertension’ OR ‘maternal syndrome’ OR ‘pregnancy-specific disorder’ OR ‘PE’ OR ‘hypertension in pregnancy’ OR ‘eclampsia’ OR ‘HELPP syndrome’ OR ‘haemolysis, elevated liver enzymes, low platelets’ OR ‘EPH’ OR ‘pregnancy toxemia’ OR ‘edema-proteinuria-hypertension gestos’) and 2023 or 2022 or 2021 (Publication Years) and Article (Document Types) and English (Languages): 13,065 results  #2: (TI=(‘cardiovascular disease’ OR ‘CVD’ OR ‘Cardiovascular disease’ OR ‘cardiovascular’ OR ‘heart disease’ OR ‘cardiometabolic risk’ OR ‘stroke’ OR ‘hypertension’ OR ‘dyslipidemia’ OR ‘dyslipidaemia’ OR ‘coronary heart disease’ OR ‘cardiomyopathy’ OR ‘congenital heart defects’ OR ‘congenital heart disease’ OR ‘heart arrhythmias’ OR ‘atherosclerotic heart diseases’ OR ‘rheumatic heart disease’ OR ‘cardiac arrhythmias’ OR ‘cerebrovascular disease’ OR ‘coronary artery disease’ OR ‘heart failure’ OR ‘heart valve disease’ OR ‘ischaemic heart disease’ OR ‘ischemic heart disease’ OR ‘atherosclerosis’ OR ‘heart attack’ OR ‘inflammatory heart disease’ OR ‘heart disorder’ OR ‘myocardial’ OR ‘ventricular arrhythmia’ OR ‘cardiac arrest’ OR ‘abnormal heart rhythms’ OR ‘hypertensive heart disease’ OR ‘carditis’ OR ‘peripheral artery disease’ OR ‘thromboembolic disease’ OR ‘myocardial infarction’ OR ‘venous thrombosis’ OR ‘acute coronary syndrome’ OR ‘cardiac failure’ OR ‘acute coronary syndrome’ OR ‘left ventricular systolic dysfunction’ OR ‘venous thromboembolism’ OR ‘cerebrovascular accident’ OR ‘deep vein thrombosis’)) OR AB=(‘cardiovascular disease’ OR ‘CVD’ OR ‘Cardiovascular disease’ OR ‘cardiovascular’ OR ‘heart disease’ OR ‘cardiometabolic risk’ OR ‘stroke’ OR ‘hypertension’ OR ‘dyslipidemia’ OR ‘dyslipidaemia’ OR ‘coronary heart disease’ OR ‘cardiomyopathy’ OR ‘congenital heart defects’ OR ‘congenital heart disease’ OR ‘heart arrhythmias’ OR ‘atherosclerotic heart diseases’ OR ‘rheumatic heart disease’ OR ‘cardiac arrhythmias’ OR ‘cerebrovascular disease’ OR ‘coronary artery disease’ OR ‘heart failure’ OR ‘heart valve disease’ OR ‘ischaemic heart disease’ OR ‘ischemic heart disease’ OR ‘atherosclerosis’ OR ‘heart attack’ OR ‘inflammatory heart disease’ OR ‘heart disorder’ OR ‘myocardial’ OR ‘ventricular arrhythmia’ OR ‘cardiac arrest’ OR ‘abnormal heart rhythms’ OR ‘hypertensive heart disease’ OR ‘carditis’ OR ‘peripheral artery disease’ OR ‘thromboembolic disease’ OR ‘myocardial infarction’ OR ‘venous thrombosis’ OR ‘acute coronary syndrome’ OR ‘cardiac failure’ OR ‘acute coronary syndrome’ OR ‘left ventricular systolic dysfunction’ OR ‘venous thromboembolism’ OR ‘cerebrovascular accident’ OR ‘deep vein thrombosis’) and 2021 or 2022 or 2023 (Publication Years) and Article (Document Types) and English (Languages): 176,749 results  #3: ALL=(‘genetic’ OR ‘candidate gene studies’ OR ‘association analysis’ OR ‘linkage studies’ OR ‘GWAS’ OR ‘Genome-wide association studies’ OR ‘Genome-wide association study’ OR ‘GWLS’ OR ‘Genome-wide linkage studies’ OR ‘gene-gene interactions’ OR ‘gene-environmental interactions’ OR ‘epistasis’ OR ‘heritability’ ) and 2021 or 2022 or 2023 (Publication Years) and Article (Document Types) and English (Languages): 339,093 results  #4: ALL=(‘DNA methylation’ OR ‘epigenetics’ OR ‘microRNA’ OR ‘histone modification’ OR ‘chromatin modification’ OR ‘histones’ OR ‘epigenetic modification’ OR ‘epigenetic mechanism’ OR ‘EWAS’ OR ‘Epigenome-wide association studies’ OR ‘Epigenome-wide DNA methylation studies’ OR ‘Epigenome-wide association study’ OR ‘non-coding RNAs’ OR ‘translational regulation’ OR ‘posttranslational regulation’ OR ‘epigenetic changes’ OR ‘transcriptional gene silencing’ OR ‘posttranslational modification’ OR ‘nucleosome remodelling’ OR ‘nucleosome remodeling’ OR ‘non-coding RNA regulation’ OR ‘RNA editing’ ) and 2021 or 2022 or 2023 (Publication Years) and Article (Document Types) and English (Languages): 57,069 results  #5: ALL=(‘shared’ OR ‘associated’ OR ‘related’ OR ‘colocalisation’ OR ‘colocalisation’ OR ‘relation’ OR ‘correlated’ OR ‘association’ OR ‘correlation’ OR ‘link’ OR ‘connected’ OR ‘connecting’ OR ‘involved’ OR ‘involving’ OR ‘combined’ OR ‘combining’ OR ‘later-life’ OR ‘later life’ OR ‘later in life’ OR ‘later risk’ OR ‘future risk’ OR ‘long term’ OR ‘later-risk’ ) and 2021 or 2022 or 2023 (Publication Years) and Article (Document Types) and English (Languages): 2,393,981 results  #1 AND #2 AND (#3 OR #4) AND #5: 771 RESULTS |
| **PUBMED**  #1: ‘preeclampsia’[Title/Abstract] OR ‘pre-eclampsia’[Title/Abstract] OR ‘Preeclampsia’[Title/Abstract] OR ‘Pre-eclampsia’[Title/Abstract] OR ‘pregnancy complication’[Title/Abstract] OR ‘pregnancy-induced hypertension’[Title/Abstract] OR ‘hypertensive disorders of pregnancy’[Title/Abstract] OR ‘toxaemia’[Title/Abstract] OR ‘toxemia’[Title/Abstract] OR ‘maternal hypertension’[Title/Abstract] OR ‘maternal syndrome’[Title/Abstract] OR ‘pregnancy-specific disorder’[Title/Abstract] OR ‘PE’[Title/Abstract] OR ‘hypertension in pregnancy’[Title/Abstract] OR ‘eclampsia’[Title/Abstract] OR ‘HELPP syndrome’[Title/Abstract] OR ‘haemolysis, elevated liver enzymes, low platelets’[Title/Abstract] OR ‘EPH’[Title/Abstract] OR ‘pregnancy toxemia’[Title/Abstract] OR ‘edema-proteinuria-hypertension gestos’[Title/Abstract] Filters: Classical Article, Clinical Study, Comparative Study, Evaluation Study, Multicenter Study, Observational Study, Twin Study, Validation Study, Humans, English, from 2021 – 2023: 122  #2: ‘cardiovascular disease’[Title/Abstract] OR ‘CVD’[Title/Abstract] OR ‘Cardiovascular disease’[Title/Abstract] OR ‘cardiovascular’[Title/Abstract] OR ‘heart disease’[Title/Abstract] OR ‘cardiometabolic risk’[Title/Abstract] OR ‘stroke’[Title/Abstract] OR ‘hypertension’[Title/Abstract] OR ‘dyslipidemia’[Title/Abstract] OR ‘dyslipidaemia’[Title/Abstract] OR ‘coronary heart disease’[Title/Abstract] OR ‘cardiomyopathy’[Title/Abstract] OR ‘congenital heart defects’[Title/Abstract] OR ‘congenital heart disease’[Title/Abstract] OR ‘heart arrhythmias’[Title/Abstract] OR ‘atherosclerotic heart diseases’[Title/Abstract] OR ‘rheumatic heart disease’[Title/Abstract] OR ‘cardiac arrhythmias’[Title/Abstract] OR ‘cerebrovascular disease’[Title/Abstract] OR ‘coronary artery disease’[Title/Abstract] OR ‘heart failure’[Title/Abstract] OR ‘heart valve disease’[Title/Abstract] OR ‘ischaemic heart disease’[Title/Abstract] OR ‘ischemic heart disease’[Title/Abstract] OR ‘atherosclerosis’[Title/Abstract] OR ‘heart attack’[Title/Abstract] OR ‘inflammatory heart disease’[Title/Abstract] OR ‘heart disorder’[Title/Abstract] OR ‘myocardial’[Title/Abstract] OR ‘ventricular arrhythmia’[Title/Abstract] OR ‘cardiac arrest’[Title/Abstract] OR ‘abnormal heart rhythms’[Title/Abstract] OR ‘hypertensive heart disease’[Title/Abstract] OR ‘carditis’[Title/Abstract] OR ‘peripheral artery disease’[Title/Abstract] OR ‘thromboembolic disease’[Title/Abstract] OR ‘myocardial infarction’[Title/Abstract] OR ‘venous thrombosis’[Title/Abstract] OR ‘acute coronary syndrome’[Title/Abstract] OR ‘cardiac failure’[Title/Abstract] OR ‘left ventricular systolic dysfunction’[Title/Abstract] OR ‘venous thromboembolism’[Title/Abstract] OR ‘cerebrovascular accident’[Title/Abstract] OR ‘deep vein thrombosis’[Title/Abstract] Filters: Classical Article, Clinical Study, Comparative Study, Evaluation Study, Multicenter Study, Observational Study, Twin Study, Validation Study, Humans, English, from 2021 – 2023: 20,287  #3: ‘genetic’ OR ‘genomics’ OR ‘epigenomics’ OR ‘DNA methylation’ OR ‘epigenetics’ OR ‘microRNA’ OR ‘histone modification’ OR ‘candidate gene studies’ OR ‘association analysis’ OR ‘linkage studies’ OR ‘chromatin modification’ OR ‘histones’ OR ‘epigenetic modification’ OR ‘epigenetic mechanism’ OR ‘GWAS’ OR ‘Genome-wide association studies’ OR ‘Genome-wide association study’ OR ‘GWLS’ OR ‘Genome-wide linkage studies’ OR ‘EWAS’ OR ‘Epigenome-wide association studies’ OR ‘Epigenome-wide DNA methylation studies’ OR ‘Epigenome-wide association study’ OR ‘non-coding RNAs’ OR ‘translational regulation’ OR ‘posttranslational regulation’ OR ‘epigenetic changes’ OR ‘transcriptional gene silencing’ OR ‘posttranslational modification’ OR ‘nucleosome remodelling’ OR ‘nucleosome remodeling’ OR ‘non-coding RNA regulation’ OR ‘RNA editing’ OR ‘gene-gene interactions’ OR ‘gene-environmental interactions’ OR ‘epistasis’ OR ‘heritability’ Filters: Classical Article, Clinical Study, Comparative Study, Evaluation Study, Multicenter Study, Observational Study, Twin Study, Validation Study, Humans, English, from 2021 – 2023: 36,666  #4: ‘shared’ OR ‘associated’ OR ‘related’ OR ‘colocalization’ OR ‘colocalisation’ OR ‘relation’ OR ‘correlated’ OR ‘association’ OR ‘correlation’ OR ‘link’ OR ‘connected’ OR ‘connecting’ OR ‘involved’ OR ‘involving’ OR ‘combined’ OR ‘combining’ OR ‘later-life’ OR ‘later life’ OR ‘later in life’ OR ‘later risk’ OR ‘future risk’ OR ‘long term’ OR ‘later-risk’ Filters: Classical Article, Clinical Study, Comparative Study, Evaluation Study, Multicenter Study, Observational Study, Twin Study, Validation Study, Humans, English, from 2021 – 2023: 98,191  #1 AND #2 AND #3 AND #4: 133 RESULTS |
| **EMBASE via OVID**  1 preeclampsia.ab,ti. 42497  2 "pre-eclampsia".ab,ti. 17523  3 Preeclampsia.ab,ti. 42497  4 "Pre-eclampsia".ab,ti. 17523  5 "pregnancy complication".ab,ti. 1426  6 "pregnancy-induced hypertension".ab,ti. 5823  7 "hypertensive disorders of pregnancy".ab,ti. 5302  8 toxaemia.ab,ti. 732  9 toxemia.ab,ti. 2632  10 "maternal hypertension".ab,ti. 1378  11 "maternal syndrome".ab,ti. 203  12 "pregnancy-specific disorder".ab,ti. 215  13 "hypertension in pregnancy".ab,ti. 2617  14 eclampsia.ab,ti. 24185  15 "HELPP syndrome".ab,ti. 31  16 "haemolysis, elevated liver enzymes, low platelets".ab,ti. 72  17 "pregnancy toxemia".ab,ti. 328  18 "edema-proteinuria-hypertension gestos".ab,ti. 0  19 EPH.ab,ti. 3943  20 PE.ab,ti. 78342  21 1 or 2 or 3 or 4 or 5 or 6 or 7 or 8 or 9 or 10 or 11 or 12 or 13 or 14 or 15 or 16 or 17 or 18 or 19 or 20 146336  22 limit 21 to (human and english language and yr="2021 - 2023" and article) 9218  23 "cardiovascular disease".ab,ti. 227559  24 "Cardiovascular disease".ab,ti. 227559  25 cardiovascular.ab,ti. 782550  26 "heart disease".ab,ti. 246878  27 "cardiometabolic risk".ab,ti. 10779  28 stroke.ab,ti. 471377  29 hypertension.ab,ti. 675357  30 dyslipidemia.ab,ti. 61061  31 dyslipidaemia.ab,ti. 10737  32 "coronary heart disease".ab,ti. 72896  33 cardiomyopathy.ab,ti. 128336  34 "congenital heart defects".ab,ti. 10269  35 "congenital heart disease".ab,ti. 44332  36 "heart arrhythmias".ab,ti. 314  37 "atherosclerotic heart diseases".ab,ti. 39  38 "rheumatic heart disease".ab,ti. 6326  39 "cardiac arrhythmias".ab,ti. 16155  40 "cerebrovascular disease".ab,ti. 26892  41 "coronary artery disease".ab,ti. 152230  42 "heart failure".ab,ti. 337270  43 "heart valve disease".ab,ti. 1310  44 "ischaemic heart disease".ab,ti. 12997  45 "ischemic heart disease".ab,ti. 41892  46 "atherosclerosis".ab,ti. 176060  47 "heart attack".ab,ti. 7406  48 "inflammatory heart disease".ab,ti. 300  49 "heart disorder".ab,ti. 240  50 "myocardial".ab,ti. 534786  51 "ventricular arrhythmia".ab,ti. 9950  52 "cardiac arrest".ab,ti. 66029  53 "abnormal heart rhythms".ab,ti. 88  54 "hypertensive heart disease".ab,ti. 2709  55 carditis.ab,ti. 2223  56 "peripheral artery disease".ab,ti. 11778  57 "thromboembolic disease".ab,ti. 6368  58 "myocardial infarction".ab,ti. 302381  59 "venous thrombosis".ab,ti. 43038  60 "acute coronary syndrome".ab,ti. 49918  61 "cardiac failure".ab,ti. 18329  62 "acute coronary syndrome".ab,ti. 49918  63 "left ventricular systolic dysfunction".ab,ti. 6224  64 "venous thromboembolism".ab,ti. 42146  65 "cerebrovascular accident".ab,ti. 8262  66 "deep vein thrombosis".ab,ti. 30463  67 23 or 24 or 25 or 26 or 27 or 28 or 29 or 30 or 31 or 32 or 33 or 34 or 35 or 36 or 37 or 38 or 39 or 40 or 41 or 42 or 43 or 44 or 45 or 46 or 47 or 48 or 49 or 50 or 51 or 52 or 53 or 54 or 55 or 56 or 57 or 58 or 59 or 60 or 61 or 62 or 63 or 64 or 65 or 66 2758236  68 limit 67 to (human and english language and yr="2021 - 2023" and article) 148589  69 "genetic*".af. 3775799  70 genomic*.af. 771748  71 "epigenomic*".af. 14804  72 "DNA methylation".af. 113040  73 "epigenetic*".af. 195605  74 microRNA.af. 228664  75 "histone modification".af. 22601  76 "candidate gene stud* ".af. 1906  77 "association analysis".af. 12688  78 "linkage stud* ".af. 7355  79 "chromatin modification".af. 2466  80 histones.af. 22882  81 "epigenetic modification".af. 8561  82 "epigenetic mechanism".af. 4035  83 GWAS.af. 34429  84 "Genome-wide association studies".af. 30057  85 "Genome-wide association study".af. 53621  86 GWLS.af. 68  87 "Genome-wide linkage study".af. 196  88 "Genome-wide linkage studies".af. 227  89 GWLS.af. 68  90 EWAS.af. 1122  91 "Epigenome-wide association studies".af. 646  92 "Epigenome-wide DNA methylation stud* ".af. 14  93 "Epigenome-wide association study".af. 923  94 "non-coding RNAs".af. 30595  95 "translational regulation".af. 5576  96 "posttranslational regulation".af. 987  97 "epigenetic changes".af. 13323  98 "transcriptional gene silencing".af. 1927  99 "posttranslational modification".af. 8614  100 "nucleosome remodelling".af. 221  101 "nucleosome remodeling".af. 1322  102 "non-coding RNA regulation".af. 93  103 "gene-gene interactions".af. 2366  104 "gene-environmental interactions".af. 356  105 epistasis.af. 9887  106 heritability.af. 32689  107 69 or 70 or 71 or 72 or 73 or 74 or 75 or 76 or 77 or 78 or 79 or 80 or 81 or 82 or 83 or 84 or 85 or 86 or 87 or 88 or 89 or 90 or 91 or 92 or 93 or 94 or 95 or 96 or 97 or 98 or 99 or 100 or 101 or 102 or 103 or 104 or 105 or 106 4333498  108 limit 107 to (human and english language and yr="2021 - 2023" and article) 220876  109 shared.af. 241270  110 associated.af. 6258659  111 related.af. 4145531  112 colocalization.af. 20041  113 colocalisation.af. 996  114 relation.af. 1177650  115 correlated.af. 1228344  116 association.af. 4614357  117 correlation.af. 1627095  118 link.af. 560116  119 connected.af. 149922  120 connecting.af. 45664  121 involved.af. 1836861  122 involving.af. 623683  123 combined.af. 1248902  124 combining.af. 264714  125 "later-life".af. 17651  126 "later life".af. 17651  127 "later in life".af. 19042  128 "later risk".af. 688  129 "future risk".af. 5922  130 "long term".af. 1429780  131 "later-risk".af. 688  132 "future risk".af. 5922  133 "long term".af. 1429780  134 "later-risk".af. 688  135 109 or 110 or 111 or 112 or 113 or 114 or 115 or 116 or 117 or 118 or 119 or 120 or 121 or 122 or 123 or 124 or 125 or 126 or 127 or 128 or 129 or 130 or 131 or 132 or 133 or 134 16798827  136 limit 135 to (human and english language and yr="2021 - 2023" and article) 1009183  137 22 and 68 and 108 and 136 : 228 RESULTS |
| **Summary:**  Scopus: 663 RESULTS  Web of Science: 771 RESULTS  PubMed: 133 RESULTS  EMBASE via OVID: 228 RESULTS  Total: 1795 RESULTS |

**SUPPLEMENTARY TABLE 5: UPDATED SEARCH (28/07/2023)**

| **SCOPUS**  #1: TITLE ( "preeclampsia" OR "pre-eclampsia" OR "pregnancy complication" OR "pregnancy-induced hypertension" OR "hypertensive disorders of pregnancy" OR "toxaemia" OR "toxemia" OR "maternal hypertension" OR "maternal syndrome" OR "pregnancy-specific disorder" OR "pe" OR "hypertension in pregnancy" OR "eclampsia" OR "helpp syndrome" OR "haemolysis, elevated liver enzymes, low platelets" OR "eph" OR "pregnancy toxemia" OR "edema-proteinuria-hypertension gestos" ) OR ABS ( "preeclampsia" OR "pre-eclampsia" OR "pregnancy complication" OR "pregnancy-induced hypertension" OR "hypertensive disorders of pregnancy" OR "toxaemia" OR "toxemia" OR "maternal hypertension" OR "maternal syndrome" OR "pregnancy-specific disorder" OR "pe" OR "hypertension in pregnancy" OR "eclampsia" OR "helpp syndrome" OR "haemolysis, elevated liver enzymes, low platelets" OR "eph" OR "pregnancy toxemia" OR "edema-proteinuria-hypertension gestos" ) AND ( LIMIT-TO ( PUBYEAR , 2023 ) ) AND ( LIMIT-TO ( DOCTYPE , "ar" ) ) AND ( LIMIT-TO ( LANGUAGE , "English" ) ) AND ( LIMIT-TO ( SRCTYPE , "j" ) : 5563 results  #2: TITLE ( "cardiovascular disease" OR "CVD" OR "Cardiovascular disease" OR "cardiovascular" OR "heart disease" OR "cardiometabolic risk" OR "stroke" OR "hypertension" OR "dyslipidemia" OR "dyslipidemia" OR "coronary heart disease" OR "cardiomyopathy" OR "congenital heart defects" OR "congenital heart disease" OR "heart arrhythmias" OR "atherosclerotic heart diseases" OR "rheumatic heart disease" OR "cardiac arrhythmias" OR "cerebrovascular disease" OR "coronary artery disease" OR "heart failure" OR "heart valve disease" OR "ischaemic heart disease" OR "ischemic heart disease" OR "atherosclerosis" OR "heart attack" OR "inflammatory heart disease" OR "heart disorder" OR "myocardial" OR "ventricular arrhythmia" OR "cardiac arrest" OR "abnormal heart rhythms" OR "hypertensive heart disease" OR "carditis" OR "peripheral artery disease" OR "thromboembolic disease" OR "myocardial infarction" OR "venous thrombosis" OR "acute coronary syndrome" OR "cardiac failure" OR "acute coronary syndrome" OR "left ventricular systolic dysfunction" OR "venous thromboembolism" OR "cerebrovascular accident" OR "deep vein thrombosis" ) OR ABS ( "cardiovascular disease" OR "CVD" OR "Cardiovascular disease" OR "cardiovascular" OR "heart disease" OR "cardiometabolic risk" OR "stroke" OR "hypertension" OR "dyslipidemia" OR "dyslipidemia" OR "coronary heart disease" OR "cardiomyopathy" OR "congenital heart defects" OR "congenital heart disease" OR "heart arrhythmias" OR "atherosclerotic heart diseases" OR "rheumatic heart disease" OR "cardiac arrhythmias" OR "cerebrovascular disease" OR "coronary artery disease" OR "heart failure" OR "heart valve disease" OR "ischaemic heart disease" OR "ischemic heart disease" OR "atherosclerosis" OR "heart attack" OR "inflammatory heart disease" OR "heart disorder" OR "myocardial" OR "ventricular arrhythmia" OR "cardiac arrest" OR "abnormal heart rhythms" OR "hypertensive heart disease" OR "carditis" OR "peripheral artery disease" OR "thromboembolic disease" OR "myocardial infarction" OR "venous thrombosis" OR "acute coronary syndrome" OR "cardiac failure" OR "acute coronary syndrome" OR "left ventricular systolic dysfunction" OR "venous thromboembolism" OR "cerebrovascular accident" OR "deep vein thrombosis" ) AND ( LIMIT-TO ( PUBYEAR , 2023 ) ) AND ( LIMIT-TO ( DOCTYPE , "ar" ) ) AND ( LIMIT-TO ( LANGUAGE , "English" ) ) AND ( LIMIT-TO ( SRCTYPE , "j" ) ): 51,022 results  #3: ALL ( "genetics" OR "genomics" OR "epigenomics" OR "DNA methylation" OR "epigenetics" OR "microRNA" OR "histone modification" OR "candidate gene studies" OR "association analysis" OR "linkage studies" OR "chromatin modification" OR "histones" OR "epigenetic modification" OR "epigenetic mechanism" OR "GWAS" OR "Genome-wide association studies" OR "Genome-wide association study" OR "GWLS" OR "Genome-wide linkage studies" OR "EWAS" OR "Epigenome-wide association studies" OR "Epigenome-wide DNA methylation studies" OR "Epigenome-wide association study" OR "non-coding RNAs" OR "translational regulation" OR "posttranslational regulation" OR "epigenetic changes" OR "transcriptional gene silencing" OR "posttranslational modification" OR "nucleosome remodelling" OR "nucleosome remodeling" OR "non-coding RNA regulation" OR "RNA editing" OR "gene-gene interactions" OR "gene-environmental interactions" OR "epistasis" OR "heritability" ) AND ( LIMIT-TO ( PUBYEAR , 2023 ) AND ( LIMIT-TO ( DOCTYPE , "ar" ) ) AND ( LIMIT-TO ( LANGUAGE , "English" ) ) AND ( LIMIT-TO ( SRCTYPE , "j" ) ): 194,560 results  #4: ALL ( "shared" OR "associated" OR "related" OR "colocalization" OR "colocalisation" OR "relation" OR "correlated" OR "association" OR "correlation" OR "link" OR "connected" OR "connecting" OR "involved" OR "involving" OR "combined" OR "combining" OR "later-life" OR "later life" OR "later in life" OR "later risk" OR "future risk" OR "long term" OR "later-risk" ) AND ( LIMIT-TO ( PUBYEAR , 2023 ) AND ( LIMIT-TO ( DOCTYPE , "ar" ) ) AND ( LIMIT-TO ( LANGUAGE , "English" ) ) AND ( LIMIT-TO ( SRCTYPE , "j" ) ): 1,310,176 results  #1 AND #2 AND #3 AND #4: 216 RESULTS |
| --- |
| **WEB OF SCIENCE**  #1: (TI=(‘preeclampsia’ OR ‘pre-eclampsia’ OR ‘Preeclampsia’ OR ‘Pre-eclampsia’ OR ‘pregnancy complication’ OR ‘pregnancy-induced hypertension’ OR ‘hypertensive disorders of pregnancy’ OR ‘toxaemia’ OR ‘toxemia’ OR ‘maternal hypertension’ OR ‘maternal syndrome’ OR ‘pregnancy-specific disorder’ OR ‘PE’ OR ‘hypertension in pregnancy’ OR ‘eclampsia’ OR ‘HELPP syndrome’ OR ‘haemolysis, elevated liver enzymes, low platelets’ OR ‘EPH’ OR ‘pregnancy toxemia’ OR ‘edema-proteinuria-hypertension gestos’)) OR AB=(‘preeclampsia’ OR ‘pre-eclampsia’ OR ‘Preeclampsia’ OR ‘Pre-eclampsia’ OR ‘pregnancy complication’ OR ‘pregnancy-induced hypertension’ OR ‘hypertensive disorders of pregnancy’ OR ‘toxaemia’ OR ‘toxemia’ OR ‘maternal hypertension’ OR ‘maternal syndrome’ OR ‘pregnancy-specific disorder’ OR ‘PE’ OR ‘hypertension in pregnancy’ OR ‘eclampsia’ OR ‘HELPP syndrome’ OR ‘haemolysis, elevated liver enzymes, low platelets’ OR ‘EPH’ OR ‘pregnancy toxemia’ OR ‘edema-proteinuria-hypertension gestos’) and 2023 (Publication Years) and Article (Document Types) and English (Languages): 3029 results  #2: (TI=(‘cardiovascular disease’ OR ‘CVD’ OR ‘Cardiovascular disease’ OR ‘cardiovascular’ OR ‘heart disease’ OR ‘cardiometabolic risk’ OR ‘stroke’ OR ‘hypertension’ OR ‘dyslipidemia’ OR ‘dyslipidaemia’ OR ‘coronary heart disease’ OR ‘cardiomyopathy’ OR ‘congenital heart defects’ OR ‘congenital heart disease’ OR ‘heart arrhythmias’ OR ‘atherosclerotic heart diseases’ OR ‘rheumatic heart disease’ OR ‘cardiac arrhythmias’ OR ‘cerebrovascular disease’ OR ‘coronary artery disease’ OR ‘heart failure’ OR ‘heart valve disease’ OR ‘ischaemic heart disease’ OR ‘ischemic heart disease’ OR ‘atherosclerosis’ OR ‘heart attack’ OR ‘inflammatory heart disease’ OR ‘heart disorder’ OR ‘myocardial’ OR ‘ventricular arrhythmia’ OR ‘cardiac arrest’ OR ‘abnormal heart rhythms’ OR ‘hypertensive heart disease’ OR ‘carditis’ OR ‘peripheral artery disease’ OR ‘thromboembolic disease’ OR ‘myocardial infarction’ OR ‘venous thrombosis’ OR ‘acute coronary syndrome’ OR ‘cardiac failure’ OR ‘acute coronary syndrome’ OR ‘left ventricular systolic dysfunction’ OR ‘venous thromboembolism’ OR ‘cerebrovascular accident’ OR ‘deep vein thrombosis’)) OR AB=(‘cardiovascular disease’ OR ‘CVD’ OR ‘Cardiovascular disease’ OR ‘cardiovascular’ OR ‘heart disease’ OR ‘cardiometabolic risk’ OR ‘stroke’ OR ‘hypertension’ OR ‘dyslipidemia’ OR ‘dyslipidaemia’ OR ‘coronary heart disease’ OR ‘cardiomyopathy’ OR ‘congenital heart defects’ OR ‘congenital heart disease’ OR ‘heart arrhythmias’ OR ‘atherosclerotic heart diseases’ OR ‘rheumatic heart disease’ OR ‘cardiac arrhythmias’ OR ‘cerebrovascular disease’ OR ‘coronary artery disease’ OR ‘heart failure’ OR ‘heart valve disease’ OR ‘ischaemic heart disease’ OR ‘ischemic heart disease’ OR ‘atherosclerosis’ OR ‘heart attack’ OR ‘inflammatory heart disease’ OR ‘heart disorder’ OR ‘myocardial’ OR ‘ventricular arrhythmia’ OR ‘cardiac arrest’ OR ‘abnormal heart rhythms’ OR ‘hypertensive heart disease’ OR ‘carditis’ OR ‘peripheral artery disease’ OR ‘thromboembolic disease’ OR ‘myocardial infarction’ OR ‘venous thrombosis’ OR ‘acute coronary syndrome’ OR ‘cardiac failure’ OR ‘acute coronary syndrome’ OR ‘left ventricular systolic dysfunction’ OR ‘venous thromboembolism’ OR ‘cerebrovascular accident’ OR ‘deep vein thrombosis’) and 2023 (Publication Years) and Article (Document Types) and English (Languages): 38,090 results  #3: ALL=(‘genetic’ OR ‘candidate gene studies’ OR ‘association analysis’ OR ‘linkage studies’ OR ‘GWAS’ OR ‘Genome-wide association studies’ OR ‘Genome-wide association study’ OR ‘GWLS’ OR ‘Genome-wide linkage studies’ OR ‘gene-gene interactions’ OR ‘gene-environmental interactions’ OR ‘epistasis’ OR ‘heritability’ ) and 2023 (Publication Years) and Article (Document Types) and English (Languages): 75,688 results  #4: ALL=(‘DNA methylation’ OR ‘epigenetics’ OR ‘microRNA’ OR ‘histone modification’ OR ‘chromatin modification’ OR ‘histones’ OR ‘epigenetic modification’ OR ‘epigenetic mechanism’ OR ‘EWAS’ OR ‘Epigenome-wide association studies’ OR ‘Epigenome-wide DNA methylation studies’ OR ‘Epigenome-wide association study’ OR ‘non-coding RNAs’ OR ‘translational regulation’ OR ‘posttranslational regulation’ OR ‘epigenetic changes’ OR ‘transcriptional gene silencing’ OR ‘posttranslational modification’ OR ‘nucleosome remodelling’ OR ‘nucleosome remodeling’ OR ‘non-coding RNA regulation’ OR ‘RNA editing’ ) and 2023 (Publication Years) and Article (Document Types) and English (Languages): 11,427 results  #5: ALL=(‘shared’ OR ‘associated’ OR ‘related’ OR ‘colocalisation’ OR ‘colocalisation’ OR ‘relation’ OR ‘correlated’ OR ‘association’ OR ‘correlation’ OR ‘link’ OR ‘connected’ OR ‘connecting’ OR ‘involved’ OR ‘involving’ OR ‘combined’ OR ‘combining’ OR ‘later-life’ OR ‘later life’ OR ‘later in life’ OR ‘later risk’ OR ‘future risk’ OR ‘long term’ OR ‘later-risk’ ) and 2023 (Publication Years) and Article (Document Types) and English (Languages): 548,722 results  #1 AND #2 AND (#3 OR #4) AND #5: 160 RESULTS |
| **PUBMED**  #1: ‘preeclampsia’[Title/Abstract] OR ‘pre-eclampsia’[Title/Abstract] OR ‘Preeclampsia’[Title/Abstract] OR ‘Pre-eclampsia’[Title/Abstract] OR ‘pregnancy complication’[Title/Abstract] OR ‘pregnancy-induced hypertension’[Title/Abstract] OR ‘hypertensive disorders of pregnancy’[Title/Abstract] OR ‘toxaemia’[Title/Abstract] OR ‘toxemia’[Title/Abstract] OR ‘maternal hypertension’[Title/Abstract] OR ‘maternal syndrome’[Title/Abstract] OR ‘pregnancy-specific disorder’[Title/Abstract] OR ‘PE’[Title/Abstract] OR ‘hypertension in pregnancy’[Title/Abstract] OR ‘eclampsia’[Title/Abstract] OR ‘HELPP syndrome’[Title/Abstract] OR ‘haemolysis, elevated liver enzymes, low platelets’[Title/Abstract] OR ‘EPH’[Title/Abstract] OR ‘pregnancy toxemia’[Title/Abstract] OR ‘edema-proteinuria-hypertension gestos’[Title/Abstract] Filters: Classical Article, Clinical Study, Comparative Study, Evaluation Study, Multicenter Study, Observational Study, Twin Study, Validation Study, Humans, English, from 2023/2/2 - 2023/7/28: 163 results  #2: ‘cardiovascular disease’[Title/Abstract] OR ‘CVD’[Title/Abstract] OR ‘Cardiovascular disease’[Title/Abstract] OR ‘cardiovascular’[Title/Abstract] OR ‘heart disease’[Title/Abstract] OR ‘cardiometabolic risk’[Title/Abstract] OR ‘stroke’[Title/Abstract] OR ‘hypertension’[Title/Abstract] OR ‘dyslipidemia’[Title/Abstract] OR ‘dyslipidaemia’[Title/Abstract] OR ‘coronary heart disease’[Title/Abstract] OR ‘cardiomyopathy’[Title/Abstract] OR ‘congenital heart defects’[Title/Abstract] OR ‘congenital heart disease’[Title/Abstract] OR ‘heart arrhythmias’[Title/Abstract] OR ‘atherosclerotic heart diseases’[Title/Abstract] OR ‘rheumatic heart disease’[Title/Abstract] OR ‘cardiac arrhythmias’[Title/Abstract] OR ‘cerebrovascular disease’[Title/Abstract] OR ‘coronary artery disease’[Title/Abstract] OR ‘heart failure’[Title/Abstract] OR ‘heart valve disease’[Title/Abstract] OR ‘ischaemic heart disease’[Title/Abstract] OR ‘ischemic heart disease’[Title/Abstract] OR ‘atherosclerosis’[Title/Abstract] OR ‘heart attack’[Title/Abstract] OR ‘inflammatory heart disease’[Title/Abstract] OR ‘heart disorder’[Title/Abstract] OR ‘myocardial’[Title/Abstract] OR ‘ventricular arrhythmia’[Title/Abstract] OR ‘cardiac arrest’[Title/Abstract] OR ‘abnormal heart rhythms’[Title/Abstract] OR ‘hypertensive heart disease’[Title/Abstract] OR ‘carditis’[Title/Abstract] OR ‘peripheral artery disease’[Title/Abstract] OR ‘thromboembolic disease’[Title/Abstract] OR ‘myocardial infarction’[Title/Abstract] OR ‘venous thrombosis’[Title/Abstract] OR ‘acute coronary syndrome’[Title/Abstract] OR ‘cardiac failure’[Title/Abstract] OR ‘acute coronary syndrome’[Title/Abstract] OR ‘left ventricular systolic dysfunction’[Title/Abstract] OR ‘venous thromboembolism’[Title/Abstract] OR ‘cerebrovascular accident’[Title/Abstract] OR ‘deep vein thrombosis’[Title/Abstract] Filters: Classical Article, Clinical Study, Comparative Study, Evaluation Study, Multicenter Study, Observational Study, Twin Study, Validation Study, Humans, English, from 2023/2/2 - 2023/7/28: 3030 results  #3: ‘genetic’ OR ‘genomics’ OR ‘epigenomics’ OR ‘DNA methylation’ OR ‘epigenetics’ OR ‘microRNA’ OR ‘histone modification’ OR ‘candidate gene studies’ OR ‘association analysis’ OR ‘linkage studies’ OR ‘chromatin modification’ OR ‘histones’ OR ‘epigenetic modification’ OR ‘epigenetic mechanism’ OR ‘GWAS’ OR ‘Genome-wide association studies’ OR ‘Genome-wide association study’ OR ‘GWLS’ OR ‘Genome-wide linkage studies’ OR ‘EWAS’ OR ‘Epigenome-wide association studies’ OR ‘Epigenome-wide DNA methylation studies’ OR ‘Epigenome-wide association study’ OR ‘non-coding RNAs’ OR ‘translational regulation’ OR ‘posttranslational regulation’ OR ‘epigenetic changes’ OR ‘transcriptional gene silencing’ OR ‘posttranslational modification’ OR ‘nucleosome remodelling’ OR ‘nucleosome remodeling’ OR ‘non-coding RNA regulation’ OR ‘RNA editing’ OR ‘gene-gene interactions’ OR ‘gene-environmental interactions’ OR ‘epistasis’ OR ‘heritability’ Filters: Classical Article, Clinical Study, Comparative Study, Evaluation Study, Multicenter Study, Observational Study, Twin Study, Validation Study, Humans, English, from 2023/2/2 - 2023/7/28: 5,051 results  #4: ‘shared’ OR ‘associated’ OR ‘related’ OR ‘colocalization’ OR ‘colocalisation’ OR ‘relation’ OR ‘correlated’ OR ‘association’ OR ‘correlation’ OR ‘link’ OR ‘connected’ OR ‘connecting’ OR ‘involved’ OR ‘involving’ OR ‘combined’ OR ‘combining’ OR ‘later-life’ OR ‘later life’ OR ‘later in life’ OR ‘later risk’ OR ‘future risk’ OR ‘long term’ OR ‘later-risk’ Filters: Classical Article, Clinical Study, Comparative Study, Evaluation Study, Multicenter Study, Observational Study, Twin Study, Validation Study, Humans, English, from 2023/2/2 - 2023/7/28: 15,494 results  #1 AND #2 AND #3 AND #4 Filters: Classical Article, Clinical Study, Comparative Study, Evaluation Study, Multicenter Study, Observational Study, Twin Study, Validation Study, Humans, English, from 2023/2/2 - 2023/7/28: 16 RESULTS |
| **EMBASE via OVID**  1 preeclampsia.ab,ti. 43689  2 "pre-eclampsia".ab,ti. 17947  3 Preeclampsia.ab,ti. 43689  4 "Pre-eclampsia".ab,ti. 17947  5 "pregnancy complication".ab,ti. 1497  6 "pregnancy-induced hypertension".ab,ti. 5956  7 "hypertensive disorders of pregnancy".ab,ti. 5642  8 toxaemia.ab,ti. 739  9 toxemia.ab,ti. 2650  10 "maternal hypertension".ab,ti. 1406  11 "maternal syndrome".ab,ti. 206  12 "pregnancy-specific disorder".ab,ti. 213  13 "hypertension in pregnancy".ab,ti. 2658  14 eclampsia.ab,ti. 24792  15 "HELPP syndrome".ab,ti. 31  16 "haemolysis, elevated liver enzymes, low platelets".ab,ti. 73  17 "pregnancy toxemia".ab,ti. 335  18 "edema-proteinuria-hypertension gestos".ab,ti. 0  19 EPH.ab,ti. 3973  20 PE.ab,ti. 80934  21 1 or 2 or 3 or 4 or 5 or 6 or 7 or 8 or 9 or 10 or 11 or 12 or 13 or 14 or 15 or 16 or 17 or 18 or 19 or 20 150582  22 limit 21 to (human and english language and yr="2023" and article) 2947  23 "cardiovascular disease".ab,ti. 233334  24 "Cardiovascular disease".ab,ti. 233334  25 cardiovascular.ab,ti. 802799  26 "heart disease".ab,ti. 252103  27 "cardiometabolic risk".ab,ti. 11203  28 stroke.ab,ti. 483959  29 hypertension.ab,ti. 692244  30 dyslipidemia.ab,ti. 63126  31 dyslipidaemia.ab,ti. 11053  32 "coronary heart disease".ab,ti. 74056  33 cardiomyopathy.ab,ti. 131964  34 "congenital heart defects".ab,ti. 10527  35 "heart arrhythmias".ab,ti. 321  36 "atherosclerotic heart diseases".ab,ti. 39  37 "rheumatic heart disease".ab,ti. 6473  38 "congenital heart defects".ab,ti. 10527  39 "cardiac arrhythmias".ab,ti. 16500  40 "cerebrovascular disease".ab,ti. 27494  41 "coronary artery disease".ab,ti. 155548  42 "heart failure".ab,ti. 346840  43 "heart valve disease".ab,ti. 1347  44 "ischaemic heart disease".ab,ti. 13215  45 "ischemic heart disease".ab,ti. 42811  46 "atherosclerosis".ab,ti. 178964  47 "heart attack".ab,ti. 7563  48 "inflammatory heart disease".ab,ti. 308  49 "heart disorder".ab,ti. 247  50 "myocardial".ab,ti. 544094  51 "ventricular arrhythmia".ab,ti. 10216  52 "cardiac arrest".ab,ti. 67992  53 "abnormal heart rhythms".ab,ti. 95  54 "hypertensive heart disease".ab,ti. 2803  55 carditis.ab,ti. 2261  56 "peripheral artery disease".ab,ti. 12211  57 "thromboembolic disease".ab,ti. 6474  58 "myocardial infarction".ab,ti. 307810  59 "venous thrombosis".ab,ti. 43892  60 "acute coronary syndrome".ab,ti. 51291  61 "cardiac failure".ab,ti. 18571  62 "acute coronary syndrome".ab,ti. 51291  63 "left ventricular systolic dysfunction".ab,ti. 6339  64 "venous thromboembolism".ab,ti. 43398  65 "cerebrovascular accident".ab,ti. 8500  66 "deep vein thrombosis".ab,ti. 31224  67 23 or 24 or 25 or 26 or 27 or 28 or 29 or 30 or 31 or 32 or 33 or 34 or 35 or 36 or 37 or 38 or 39 or 40 or 41 or 42 or 43 or 44 or 45 or 46 or 47 or 48 or 49 or 50 or 51 or 52 or 53 or 54 or 55 or 56 or 57 or 58 or 59 or 60 or 61 or 62 or 63 or 64 or 65 or 66 2821225  68 limit 67 to (human and english language and yr="2023" and article) 45029  69 "genetic*".af. 3884083  70 genomic*.af. 800882  71 "epigenomic*".af. 15308  72 "DNA methylation".af. 118023  73 "epigenetic*".af. 203216  74 microRNA.af. 236668  75 "histone modification".af. 23653  76 "candidate gene stud* ".af. 1911  77 "association analysis".af. 13164  78 "linkage stud* ".af. 7459  79 "chromatin modification".af. 2478  80 histones.af. 23169  81 "epigenetic modification".af. 9601  82 "epigenetic mechanism".af. 4126  83 GWAS.af. 36233  84 "Genome-wide association studies".af. 31208  85 "Genome-wide association study".af. 58193  86 GWLS.af. 71  87 "Genome-wide linkage study".af. 197  88 "Genome-wide linkage studies".af. 235  89 EWAS.af. 1205  90 "Epigenome-wide association studies".af. 685  91 "Epigenome-wide DNA methylation stud* ".af. 14  92 "Epigenome-wide association study".af. 985  93 "non-coding RNAs".af. 31756  94 "translational regulation".af. 5639  95 "posttranslational regulation".af. 992  96 "transcriptional gene silencing".af. 1960  97 "posttranslational modification".af. 8736  98 "nucleosome remodelling".af. 229  99 "nucleosome remodeling".af. 1348  100 "non-coding RNA regulation".af. 108  101 "gene-gene interactions".af. 2410  102 "gene-environmental interactions".af. 360  103 epistasis.af. 10119  104 heritability.af. 33574  105 "epigenetic changes".af. 13756  106 69 or 70 or 71 or 72 or 73 or 74 or 75 or 76 or 77 or 78 or 79 or 80 or 81 or 82 or 83 or 84 or 85 or 86 or 87 or 88 or 89 or 90 or 91 or 92 or 93 or 94 or 95 or 96 or 97 or 98 or 99 or 100 or 101 or 102 or 103 or 104 or 105 4456386  107 limit 106 to (human and english language and yr="2023" and article) 66065  108 shared.af. 253126  109 associated.af. 6430509  110 related.af. 4275859  111 colocalization.af. 20478  112 colocalisation.af. 1027  113 relation.af. 1199448  114 correlated.af. 1256523  115 association.af. 4763103  116 correlation.af. 1679333  117 link.af. 578445  118 connected.af. 154650  119 connecting.af. 47213  120 involved.af. 1881917  121 involving.af. 639660  122 combined.af. 1286468  123 combining.af. 275307  124 "later-life".af. 18130  125 "later life".af. 18130  126 "later in life".af. 19545  127 "later risk".af. 684  128 "future risk".af. 6109  129 "long term".af. 1468659  130 "later-risk".af. 684  131 "future risk".af. 6109  132 "long term".af. 1468659  133 "later-risk".af. 684  134 108 or 109 or 110 or 111 or 112 or 113 or 114 or 115 or 116 or 117 or 118 or 119 or 120 or 121 or 122 or 123 or 124 or 125 or 126 or 127 or 128 or 129 or 130 or 131 or 132 or 133 17246243  135 limit 134 to (human and english language and yr="2023" and article) 311558  136 22 and 68 and 107 and 135: 89 RESULTS |
| **Summary:**  Scopus: 216 RESULTS  Web of Science: 160 RESULTS  PubMed: 16 RESULTS  EMBASE via OVID: 89 RESULTS  Total: 481 RESULTS |

| **SUPPLEMENTARY TABLE 6: Quality Assessment Scores using the Newcastle-Ottawa Scale for Case-control Studies** | | | | | | | | | | |
| --- | --- | --- | --- | --- | --- | --- | --- | --- | --- | --- |
| **Author, Year** | **Adequate definition of cases** | **Representativeness of the cases** | **Selection of controls** | **Definition of controls** | **Comparability** | | **Ascertainment of exposure** | **Same method of ascertainment for cases and controls** | **Non-response rate** | **Total score, Decision and reason** |
|  |  |  |  |  | **Cases and controls**  **matched in design** | **Confounders adjusted in the analysis** |  |  |  |  |
| **A. Johansson, 2011^(37)^** | 1 | 1 | 1 | 1 | 0 | 0 | 1 | 1 | 0 | **6/9, Excluded:**  Studied shared genetic risk factors of PE and CVD using two different cohorts of PE and CVD |
| **L.G Best, 2013^(40)^** | **Discovery cohort:**  American Indian Population:  1 | 1 | 1 | 1 | 0.5 | 1 | 1 | 1 | 0 | **7.5/9, Excluded:** Only used IBC Cardio chip microarray to determine SNPs associated with both CVD and PE. Did not test for CVD or CVD risk factors. |
|  | **Replication cohort:**  Boston Study:  1 | 1 | 1 | 1 | 0.5 | 1 | 1 | 1 | 0 |  |
|  | **Replication cohort:**  Philadelphia study:  1 | 1 | 1 | 1 | 0.5 | 1 | 1 | 1 | 0 |  |
| **M.P. Johnson, 2013^(20)^**  **(Replication cohorts)** | Australian singletons:  1 | 1 | 1 | 1 | 0.5 | 0 | 1 | 1 | 0 | **6.5/9,**  **Excluded:** Studied shared genetic risk factors of PE and CVD using two different cohorts of PE and CVD |
|  | Norwegian singletons:  1 | 1 | 1 | 1 | 0 | 0 | 1 | 1 | 0 | **6/9,**  **Excluded:** Studied shared genetic risk factors of PE and CVD using two different cohorts of PE and CVD |
|  | Finnish singletons:  1 | 1 | 1 | 1 | 0.5 | 0 | 1 | 1 | 0 | **6.5/9,**  **Excluded:**  Studied shared genetic risk factors of PE and CVD using two different cohorts of PE and CVD |
| **A.S. Kvehaugen, 2014^(48)^** | 1 | 1 | 1 | 1 | 0.5 | 1 | 1 | 1 | 0 | **7.5/9,**  **Included** |
| **C.Oudejans, 2016^(43)^** | 1 | 1 | 1 | 1 | 0.5 | 1 | 1 | 1 | 1 | **8.5/9,**  **Included** |
| **I. Romagnuolo,2016^(44)^** | 1 | 1 | 1 | 1 | 0.5 | 1 | 1 | 1 | 0 | **7.5/9,**  **Included** |
| **Z.B. Gungor, 2018^(42)^** | 1 | 1 | 1 | 1 | 1 | 1 | 1 | 1 | 0 | **8/9, Excluded:**  Studied Lp-PLA2 levels in women with PE during the time of delivery. No follow-up on later-life CVD |
| **N.Dayan, 2018^(47)^** | 0 | 1 | 1 | 1 | 0.5 | 0 | 1 | 1 | 0 | **5.5/9,**  **Included** |
| **K.J.Gray, 2018^(39)^** | **Discovery cohort:**  Boston Hospital Collections:  1 | 1 | 0.5 | 1 | 0.5 | 0.5 | 1 | 1 | 0 | **6.5/9,**  **Excluded:**  Only used Cardio chip to determine SNPs associated between CVD and PE, used predisposed CVD genes: did not test for CVD. |
|  | **Discovery cohort:**  University of Southern California (USC) Collections:  1 | 0.5 | 1 | 1 | 0.5 | 0.5 | 1 | 1 | 0 | **7/9,**  **Excluded:**  Only used Cardio chip to determine SNPs associated between CVD and PE, did not test for CVD. |
|  | **Discovery cohort:**  Children’s Hospital of Philadelphia Collection:  1 | 1 | 1 | 1 | 0.5 | 0.5 | 1 | 1 | 0 | **7.5/9,**  **Excluded:**  Only used Cardio chip to determine SNPs associated between CVD and PE, did not test for CVD. |
|  | **Discovery cohort:**  Yale-New Haven Hospital Collection:  1 | 1 | 1 | 1 | 1 | 0.5 | 1 | 1 | 0 | **7.5/9,**  **Excluded:**  Only used Cardio chip to determine SNPs associated between CVD and PE, used predisposed CVD genes: did not test for CVD. |
|  | **Discovery cohort:**  University of Iowa Hospitals and Clinics Collection:  1 | 1 | 0 | 0 | 0 | 1 | 1 | 1 | 0 | **5/9,**  **Excluded:**  Only used Cardio chip to determine SNPs associated between CVD and PE, used predisposed CVD genes: did not test for CVD. |
|  | **Replication cohort:**  SOPHIA Study:  1 | 1 | 1 | 1 | 0.5 | 1 | 1 | 1 | 0 | **7.5/9,**  **Excluded:**  Only used Cardio chip to determine SNPs associated between CVD and PE, used predisposed CVD genes: did not test for CVD. |
|  | **Replication cohort:**  Inova Health System Study:  1 | 1 | 1 | 1 | 0 | 1 | 1 | 1 | 0 | **7/9,**  **Excluded:**  Only used Cardio chip to determine SNPs associated between CVD and PE, used predisposed CVD genes: did not test for CVD. |
| **K. Schlosser, 2020^(46)^** | Cohort 1:  0 | 1 | 1 | 1 | 1 | 0 | 1 | 1 | 0 | **6/9,**  **Included** |
|  | Cohort 2:  0.5 | 1 | 1 | 1 | 1 | 0 | 1 | 1 | 0 | **6.5/9,**  **Included** |
| **A. Tuten, 2021^(41)^** | 1 | 1 | 1 | 1 | 1 | 1 | 1 | 1 | 0 | **8/9,**  **Excluded:**  Studied Lp-PLA2 levels in women with PE during the time of delivery. No follow-up on later-life CVD. |
| **I.V. da Silva, 2021^(45)^** | 1 | 1 | 1 | 1 | 0.5 | 1 | 1 | 1 | 0 | **7.5/9,**  **Included** |

| SUPPLEMENTARY TABLE 7: Quality Assessment Scores using the Newcastle-Ottawa Scale for Cohort Studies | | | | | | | | | |
| --- | --- | --- | --- | --- | --- | --- | --- | --- | --- |
| Author, Year | **Representative of the exposed cohort** | **Selection of the non-exposed cohort** | **Ascertainment of exposure** | **Demonstration that the outcome of interest was not present at the start of study** | **Comparability of cohort** | **Assessment of outcome** | **Follow-up duration to capture outcomes** | **Adequacy of follow-up** | **Total score and Decision** |
| A. Johansson, 2011^(37)^ | 1 | 0 | 1 | 0 | 0 | 1 | 0 | 0 | **3/9,**  **Excluded:** Studied shared genetic risk factors of PE and CVD using two different cohorts of PE and CVD |
| M.P. Johnson, 2013 ^(20)^ | SAFHS study:  1 | 0 | 1 | 0 | 0 | 1 | 0 | 0 | **3/9,**  **Excluded:**  Studied shared genetic risk factors of PE and CVD using two different cohorts of PE and CVD |
|  | **Discovery cohort:**  AUS/NZL family:  1 | 1 | 1 | 0 | 0 | 1 | 0 | 0 | **4/9,**  **Excluded:**  Studied shared genetic risk factors of PE and CVD using two different cohorts of PE and CVD |
| M. Loset, 2014^(19)^ | 1 | 0 | 1 | 1 | 1, 0 | 1 | 1 | 0 | **6/9,**  **Excluded:**  studied shared genetic risk between PE and CVD based on two different studies. The number of women with PE not clear. |

| **SUPPLEMENTARY TABLE 8: Quality Assessment Scores using the Newcastle-Ottawa Scale for Cross-sectional Studies** | | | | | | | | |
| --- | --- | --- | --- | --- | --- | --- | --- | --- |
| **Author, Year** | **Representative of the sample** | **Sample size** | **Non-respondents** | **Ascertainment of exposure** | **Comparability of the outcome groups** | **Assessment of outcome** | **Statistical test** | **Total score and Decision** |
| **C. Fatini, 2016^(38)^** | 1 | 1 | 1 | 1,0 | 1,0 | 1,1 | 1 | **8/10,**  **Excluded:**  Studied PE and CVD relationship using two different sets of women from the same population. |

| **SUPPLEMENTARY TABLE 9: Quality Assessment using the Newcastle-Ottawa Scale for Case-control Studies (Detailed Description)** | | | | | | | | | | |
| --- | --- | --- | --- | --- | --- | --- | --- | --- | --- | --- |
| **Author, Year, PubMed ID (PMID)** | **Adequate definition of cases** | **Representativeness of the cases** | **Selection of controls** | **Definition of controls** | **Comparability** | | **Ascertainment of exposure** | **Same method of ascertain-ment for cases and controls** | **Non-response rate** | **Reason of Inclusion or Exclusion** |
|  |  |  |  |  | **Cases and controls matched in design** | **Confounders adjusted in the analysis** |  |  |  |  |
| **A. Johansson, 2011^(37)^, PMID: 21343950** | The Norwegian PE study: from medical records, PE diagnosis performed | Pregnant women delivered by caesarean section with PE | Pregnant women delivered by caesarean section without PE: same population | Pregnant women with no previous history of PE, no multiple pregnancies, no pregnancies with chromosomal aberrations, foetal and placental structural abnormalities, or suspected perinatal infections | Not matched for  sample size (1:2) or any characteristics  Cases = 37, Controls = 58 | Not adjusted | Transcriptional profiling: Illumina BeadChips | Yes | Unclear | **Excluded:** Studied shared genetic risk factors of PE and CVD using two different cohorts of PE and CVD and in two different sets of women. Did not longitudinally test for CVD following PE, or there was no follow-up study. |
| **L.G. Best, 2013^(40)^, PMID: 23940726** | **Discovery cohort:**  American Indian Population:  PE women based on a medical record database using ICD9 codes from a hospital in Belcourt, North Dakota | PE women based on diagnostic criteria | From the same hospital | Those not meeting PE diagnostic criteria | Matched for age, ethnicity, and date of delivery;  sample size (1:2) not matched;  Cases = 140, Controls = 270 | Adjusted for age, delivery, nulliparity and BMI | SNP genotyping | Yes | Unclear | **Excluded:** Only used IBC Cardio chip microarray to determine SNPs associated with both CVD and PE. Tested for *CRP* variants and their association with the risk or severity of PE. Did not test for CVD or CVD risk factors. |
|  | **Replication cohort:**  Boston Study: 3 Harvard affiliated Hospitals: PE women identified based on ICD9 codes and validated using medical records | PE women based on ACOG 2022 diagnostic criteria and physician diagnosis | From same population | Normal term pregnancies | Matched for ethnicity;  sample size (1:0) not matched;  Cases = 105, Controls = 17 | Adjusted for study site and ten principal components | SNP genotyping | Yes | Unclear |  |
|  | **Replication cohort:**  Philadelphia study: PE women based on medical records from the Children’s Hospital of Philadelphia | PE women based on diagnosis criteria and medical records | From the same population | Normotensive mothers | Matched for ethnicity;  sample size (1:5) not matched;  Cases = 73, Controls = 353 | Adjusted for study site and ten principal components | SNP genotyping | Yes | Unclear |  |
| **M.P. Johnson, 2013^(20)^, PMID: 23420841**  **(Replication cohorts)** | Australian singletons:  PE diagnosis by clinicians based on the Society of Obstetric Medicine of Australia  and New Zealand for the management of hypertensive diseases of pregnancy | Based on PE diagnosis criteria and women who were previously normotensive | From the same population to cases | Women who have had a normal pregnancy | Sample size matched (1:1 i.e., rounding 1:1.2);  Cases (N) = 499, Control (N) = 596 | Unadjusted | Genotyping and association analysis | Yes | Non- respondents not mentioned | **Excluded:**  Studied shared genetic risk factors of PE and CVD using different cohorts of PE and CVD and in different sets of women. Did not longitudinally test for CVD following PE, or there was no follow-up study. |
|  | Norwegian singletons: PE based on MBRN guidelines | Women with PE based on the diagnostic criteria | From the same population as cases | History of a normal healthy pregnancy | Sample size not matched (1:2); Cases (N) = 499, Control (N) = 596 | Unadjusted | Genotyping and association analysis | Yes | Non- respondents not mentioned |  |
|  | Finnish singletons:  FINNPEC study cohort and Southern Finnish cohort, based on PE diagnostic criteria and medical records, also validated by a clinician | Women with pre-eclamptic pregnancy and had no medical history of chronic hypertension, type 1 diabetes or renal disease | From the same population as cases | Women with no PE | Sample size matched (1:1); Cases (N) = 760, Control (N) = 759 | Unadjusted | Genotyping and association analysis | Yes | Non- respondents not mentioned |  |
| **A.S. Kvehaugen, 2014^(48)^, PMID: 24593135** | HUNT2 Survey data linked with the diagnosis of PE registered in MBRN records. | Women with a diagnosis of PE in MBRN before taking part in the HUNT2 study and those with available DNA samples at HUNT biobank. | From the same source as cases: HUNT2 survey and MBRN records | Women with no prior PE and a minimum of one normal pregnancy at least one year prior to taking part in the HUNT2 study | Sample size not matched (1:2);  Cases (N) = 934, Controls (N)= 2011; matched for age | Age, BMI and history of PE adjusted | SNP analysis: TaqMan-based genotyping | Yes | Non- respondents not mentioned | **Included:** Studied PE and later-life hypertension using the same cohort and same women. Tested for polymorphisms associated with both PE and later-life hypertension. The study was conducted 15 years after PE |
| **C.Oudejans, 2016^(43)^, PMID: 26870946** | Two monozygotic parous twin sisters with HELPP syndrome from medical records of VU Medical Centre, Amsterdam | Two monozygotic parous twin sisters with HELPP syndrome | Twin sisters, controls from the same cohort | Women with uncomplicated pregnancy or unaffected twin sisters | Only sample size matched (1:1);  Case (N) = 1, Control (N)= 1 | Adjusted for age, SBP, DBP, BMI, Waist and Hip circumference, Smoking, Medication | Epigenetic risk profiling by whole genome bisulphite sequencing | Yes | Yes, same for both groups | **Included:** studied epigenetic changes associated with increased CVD risk following PE using the same cohort and same women. Tested on PE and CVD risk factors. |
| **I. Romagnuolo,2016^(44)^, PMID: 26827667** | Women with placenta-mediated pregnancy complications: based on clinical reports of Gender Medicine Clinic, Florence | Women with placenta-mediated pregnancy complications | Controls from the same clinic | Women with no history of vascular disease or obstetric complications | Sample size matched (1:1, i.e., rounding 1: 0.75); Cases (N) = 360, Control (N) = 270;  not matched for other characteristics | Adjusted for age, familial history of CVD, hypertension, dyslipidaemia, smoking habit, BMI and timing for delivery | Genotyping  Polymorphisms: PCR | Yes | Unclear | **Included:** studied Lp(a) levels and *LPA* polymorphisms and their association with PE using the same cohort and same women. |
| **Z.B. Gungor, 2018^(42)^, PMID: 28784006** | Preeclamptic women based on medical records from Cerrahpasa Medical School, Turkey | Pregnant women with early-onset PE and late-onset PE | From the same source as cases | Pregnant women with no PE history | Matched for sample size (3:1); Cases (N) = 150, Control (N)= 50; matched for age and BMI | Adjusted for PTX3, age, DBP, TC, HDL levels | SNP genotyping using iPLEX™ assay | Yes | Unclear | **Excluded:** studied on Lp-PLA2 and various other CVD risk factors in women with PE during the time of delivery. The CVD risk was not studied following PE |
| **K.J. Gray, 2018^(39)^, PMID: 29967039** | **Discovery cohort:**  Boston Hospital Collections:  Massachusetts General Hospital and Beth Israel Deaconess Medical Center: PE diagnosis validated by physician medical record | Preeclamptic women based on medical records | Partially from the same hospital: Beth Israel Deaconess Medical Center | Normotensive women, age-matched and of European, Black and Hispanic ancestry with healthy term pregnancies | Matched for age, ethnicity; Sample sizes not matched;  European:  (1:0.3)  Cases (N) = 263, Controls (N) = 74 | Adjusted for ten principal components in European samples | GWAS and multi-ethnic assessment | Yes | Unclear | **Excluded**: only used Cardio chip to determine SNPs associated between CVD and PE, did not test for CVD. |
|  | **Discovery cohort:**  University of Southern California (USC) Collections: HELPP Syndrome Society and Los Angeles County +USC Medical Center: PE diagnosis based on medical records | PE/HELPP cases based on medical records | Controls only from one center | Normotensive women, unrelated friends of cases and their families | Matched for age, ethnicity;  Sample sizes not matched | Adjusted for 10 principal components in European samples | GWAS and multi-ethnic assessment | Yes | Unclear |  |
|  | **Discovery cohort:**  Children’s Hospital of Philadelphia Collection: PE diagnosis based on medical records | PE women based on medical records | Controls from same cohort | Normotensive women | Matched for age, ethnicity; Sample size not matched,  European:  (1:5)  Cases (N) = 69, Controls (N) = 345 | Adjusted for 10 principal components in European samples | GWAS and multi-ethnic assessment | Yes | Unclear |  |
|  | **Discovery cohort:**  Yale-New Haven Hospital Collection: PE diagnosis based on medical records | PE women based on medical records | Controls from same cohort | Normotensive women | Matched for age, ethnicity; Sample size matched,  European:  (1:3)  Cases (N) = 11, Controls (N) = 30; | Adjusted for 10 principal components in European samples | GWAS and multi-ethnic assessment | Yes | Unclear |  |
|  | **Discovery cohort:**  University of Iowa Hospitals and Clinics Collection: PE diagnosis based on medical records | PE women based on medical records | Not mentioned | Not mentioned | Not applicable, controls from different ethnicity; Cases (N) = 75 | Adjusted for 10 principal components in European samples | GWAS and multi-ethnic assessment | Yes | Unclear |  |
|  | **Replication cohort:**  SOPHIA Study: PE cases based on infant’s birth certificate and chart review | PE women based on infant’s birth certificate: mentioned if mother’s had pregnancy induced hypertension or eclampsia; charts of PE women reviewed | Controls from same cohort | Primiparous women with no indication of hypertension in infant’s birth certificate; normotensive women | Not matched for any characteristics; sample size matched (1:1); Cases (N) = 177, Controls (N)= 116 | Adjusted for 10 principal components in European samples | GWAS and multi-ethnic assessment | Yes | Unclear |  |
|  | **Replication cohort:**  Inova Health System Study: PE diagnosis reviewed by clinicians | PE women based on medical records | Controls from same cohort | Normotensive women | Not matched for any characteristics; sample size not matched (1:10); Cases (N) = 35, Controls (N)= 340 | Adjusted for 10 principal components in European samples | GWAS and multi-ethnic assessment | Yes | Unclear |  |
| **N. Dayan, 2018^(47)^, PMID: 29800045** | GENEXIS-PRAXY multicentre study of adults hospitalised with ACS and prior PE  based on self-reported questionnaires | Women with ACS and prior PE, classified as prior PE: if reported PE, hypertension and proteinuria | Same cohort as cases | Normotensive women | Sample size not matched (1:5); Cases (N) = 30, Control (N) = 146;  matched for age, hypertension, smoking status and dyslipidaemia | Unadjusted | Circulating miRNA assessment: PCR | Yes | Non- respondents not mentioned | **Included:** The study looked for miRNA patterns between PE and NT in the same women with ACS from the same cohort. |
| **K. Schlosser, 2020^(46)^, PMID: 31899480** | Cohort1:  GENEXIS-PRAXY multicentre study of adults hospitalised with ACS and prior PE  based on self-reported questionnaires | Women with ACS and with a history of PE, classified as prior PE: if reported PE, hypertension and proteinuria | Same cohort as cases | Normotensive women | Sample size matched (1:1); Cases (N) = 18, Control (N) = 17;  Matched for age, BMI, menopausal status, several CVD risks factors (hypertension, diabetes, smoking) | Unadjusted | Circulating miRNA assessment: PCR | Yes | Non- respondents not mentioned | **Included:** studied circulating miRNA levels associated with both PE and ACS using the same women from the same cohort. |
|  | Cohort 2:  COPS study, Generation Scotland: Scottish Family Health Study and the Proteomics in Preeclampsia: based on questionnaires and medical records | Women without ACS and with a history of PE | Same cohort as cases | Normotensive women | Sample size matched (1:1); Cases (N) = 20, Control (N) = 20;  Matched for age, BMI, menopausal status, several CVD risks factors (hypertension, diabetes, smoking) | Unadjusted | Circulating miRNA assessment: PCR | Yes | Non- respondents not mentioned |  |
| **A. Tuten,2021^(41)^, PMID: 31113255** | Preeclamptic women based on medical records from Cerrahpasa Medical School, Turkey | Pregnant women with early-onset PE and late-onset PE | From the same source as cases | Pregnant women with no PE history | Sample size matched (3:1); Cases (N) = 150, Control (N)= 50;  age and BMI matched | Adjusted for PTX3, age, DBP, TC, HDL levels | SNP genotyping using iPLEX™ assay | Yes | Unclear | **Excluded:** studied on Lp-PLA2 and various other CVD risk factors in women with PE during the time of delivery. The CVD risk was not studied following PE, or there was no follow-up study. |
| **I.V. da Silva, 2021^(45)^, PMID: 33652340** | Preeclamptic women based on medical records from Júlio Diniz Maternity, Maria Pia Hospital, Oporto. | Preeclampsia women: PE diagnosis based on ISSHP criteria | From same hospital as cases | Normotensive women | Sample size not matched (1:2); Cases (N) = 48,  Control (N) = 98;  Matched for age | Adjusted for age | Polymorphism genotyping by qPCR | Yes | Unclear | **Included:**  Studied polymorphisms associated with PE and later-life hypertension. A Follow-up study on later-life hypertension was conducted following PE |
| **Abbreviations:** **ACOG:** American College of Obstetricians and Gynaecologists’; **ACS:** Acute Coronary Syndrome; **BMI:** Body Mass Index; **COPS:** The Cardiovascular Consequences of Preeclampsia Study; ***CRP*:** C-Reactive Protein; **CVD:** Cardiovascular disease; **DBP:** Diastolic Blood Pressure; **FINNPEC:** Finnish Genetics of Pre-eclampsia Consortium; **GENEXIS-PRAXY:** GENdEr and Sex determInantS of cardiovascular disease: From bench to beyond-Premature Acute Coronary SYndrome; **GWAS:** Genome-Wide Association Studies; **HDL:** High-Density Lipoprotein; **HELPP:** Haemolysis, Elevated Liver enzymes and Low Platelets; **HUNT2:** phase 2 of the Nord-Trondelag Health Study; **IBC:** ITMAT-Broad-CARe array; **ICD-9:** International Classification of Diseases-Ninth Revision**; ISSHP:** International Society for the Study of Hypertension in Pregnancy; **Lp(a):** Lipoprotein(a); **Lp-PLA2:** Lipoprotein-associated phospholipase A2; **MBRN:** Medical Birth Registry of Norway; **PCR:** Polymerase Chain Reaction; **PE:** Preeclampsia; **PTX3:** Pentraxin-3; **qPCR:** Real-Time Quantitative Polymerase Chain Reaction; **SBP:** Systolic Blood Pressure; **SNP:** Single Nucleotide Polymorphism; **SOPHIA:** Study of Pregnancy Hypertension in Iowa; **TC:** Total Cholesterol | | | | | | | | | | |

| **SUPPLEMENTARY TABLE 10: Quality Assessment using the Newcastle-Ottawa Scale for Cohort Studies (Detailed Description)** | | | | | | | | | |
| --- | --- | --- | --- | --- | --- | --- | --- | --- | --- |
| **Author, Year, PubMed ID (PMID)** | **Representative of the exposed cohort** | **Selection of the non-exposed cohort** | **Ascertainment of exposure** | **Demonstration that the outcome of interest was not present at the start of the study** | **Comparability of cohort** | **Assessment of outcome** | **Follow-up duration to capture outcomes** | **Adequacy of follow-up** | **Reason of Inclusion or Exclusion** |
| **A. Johansson, 2011^(37)^, PMID: 21343950** | The SAFHS study:  Individuals from extended families of Mexican Americans; with CVD | Not mentioned | Transcriptional profiling | Not mentioned | Not mentioned | The SAFHS study: CVD phenotype assessments (serum levels of lipids, lipoproteins, glucose, hormones, adiposity, blood pressure) | No follow-up | Not relevant | **Excluded:**  Studied shared genetic risk factors of PE and CVD using two different cohorts of PE and CVD and in two different sets of women. Did not longitudinally test for CVD following PE, or there was no follow-up study. |
| **M.P. Johnson, 2013**^(20)^**, PMID: 23420841** | The SAFHS study:  extended families of Mexican Americans with CVD | Not mentioned | Transcriptional profiling and genotyping | Not mentioned | Not mentioned | The SAFHS study: CVD phenotype assessments | Not relevant | Not relevant | **Excluded:**  Studied shared genetic risk factors of PE and CVD using different cohorts of PE and CVD and in different sets of women. Did not longitudinally test for CVD following PE, or there was no follow-up study. |
|  | **Discovery cohort:**  Australian/ New Zealand families: PE diagnosis by clinicians based on the Australasian Society for the Study of Hypertension in Pregnancy criteria | Unaffected: normal pregnancies | Genotyping and association analysis | Unclear | Not matched for any characteristics | PE diagnosis by clinical assessments and based on diagnostic criteria | Not relevant | Not relevant |  |
| **M. Loset, 2014**^(19)^**, PMID: 26104425** | Pregnant women with PE or a history of PE | Not mentioned | SNP genotyping and association analysis | Yes | Not matched for any characteristics; adjusted for sex and maternal age | CVD risk factors: Assessment of SBP, DBP, height, weight, waist-hip ratio, abdominal skinfold, insulin, glucose, TC, HDL-C, LDL-C, TG | Yes, after eight years | 41.9% lost to follow up | **Excluded:** studied shared genetic risk between PE and CVD based on two different studies; mainly focused on the shared genetic risk in children. The number of PE women is not clear. |
| **Abbreviations: CVD:** Cardiovascular disease; **DBP:** Diastolic Blood Pressure; **HDL-C:**  High-Density Lipoprotein-Cholesterol; **LDL-C:** Low-Density Lipoprotein-Cholesterol; **PE**: Preeclampsia; **SAFHS:** San Antonio Family Heart Study; **SNP:** Single Nucleotide Polymorphism; **TC:** Total cholesterol; **TG:** Triglycerides | | | | | | | | | |

| **SUPPLEMENTARY TABLE 11: Quality Assessment using the Newcastle-Ottawa Scale for Cross-sectional Studies (Detailed Description)** | | | | | | | | |
| --- | --- | --- | --- | --- | --- | --- | --- | --- |
| **Author, Year, PubMed ID (PMID)** | **Representative of the sample** | **Sample size** | **Non-respondents** | **Ascertainment of exposure** | **Comparability of the outcome groups** | **Assessment of outcome** | **Statistical test** | **Reason of Inclusion or Exclusion** |
| **C. Fatini, 2016**^(38)^ | Entire population from the Gender Medicine Clinic of the Center for Atherothrombotic disease | Satisfactory (at least 270 subjects for each group) | 19/1406 women did not respond ~ 1.3% non-response rate | SNP genotyping: TaqMan assay | Matched for age | Premature CVD: unclear  Late pregnancy complications: gynaecologists’ clinical report | Chi-square test  Odds ratios with 95% CI | **Excluded:**  Studied PE and CVD relationship using two different sets of women from the same population. |
| **Abbreviations: ACS:** Acute Coronary Syndrome; **CVD:** Cardiovascular disease; **PE:** Preeclampsia; **SNP:** Single Nucleotide Polymorphism | | | | | | | | |
